# Pharmacological depletion of microglia alleviates neuronal and vascular damage in the diabetic CX3CR1-WT retina but not in CX3CR1-KO or hCX3CR1^I249/M280^-expressing retina

**DOI:** 10.3389/fimmu.2023.1130735

**Published:** 2023-03-22

**Authors:** Kaira A. Church, Derek Rodriguez, Andrew S. Mendiola, Difernando Vanegas, Irene L. Gutierrez, Ian Tamayo, Abdul Amadu, Priscila Velazquez, Sandra M. Cardona, Stefka Gyoneva, Anne C. Cotleur, Richard M. Ransohoff, Tejbeer Kaur, Astrid E. Cardona

**Affiliations:** ^1^ Department of Molecular Microbiology and Immunology, The University of Texas at San Antonio, San Antonio, TX, United States; ^2^ South Texas Center for Emerging Infectious Diseases, The University of Texas at San Antonio, San Antonio, TX, United States; ^3^ Department of Pharmacology and Toxicology, Universidad Complutense de Madrid, Centro de Investigacion Biomedica en Red Salud Mental (CIBERSAM), Madrid, Spain; ^4^ Human Genetics, Cerevel Therapeutics, Cambridge, MA, United States; ^5^ Acute Neurology, Biogen, Cambridge, MA, United States; ^6^ Department of Neurosciences, The Cleveland Clinic Lerner Research Institute, Cleveland, OH, United States; ^7^ Neuroinflammation Research Center, The Cleveland Clinic Lerner Research Institute, Cleveland, OH, United States; ^8^ Biomedical Sciences, School of Medicine, Creighton University, Omaha, NE, United States

**Keywords:** microglia, depletion, CX3CR1 chemokine receptor, diabetic retinopathy, inflammation

## Abstract

Diabetic retinopathy, a microvascular disease characterized by irreparable vascular damage, neurodegeneration and neuroinflammation, is a leading complication of diabetes mellitus. There is no cure for DR, and medical interventions marginally slow the progression of disease. Microglia-mediated inflammation in the diabetic retina is regulated *via* CX3CR1-FKN signaling, where FKN serves as a calming signal for microglial activation in several neuroinflammatory models. Polymorphic variants of *CX3CR1*, *hCX3CR1^I249/M280^*, found in 25% of the human population, result in a receptor with lower binding affinity for FKN. Furthermore, disrupted CX3CR1-FKN signaling in *CX3CR1*-KO and *FKN*-KO mice leads to exacerbated microglial activation, robust neuronal cell loss and substantial vascular damage in the diabetic retina. Thus, studies to characterize the effects of *hCX3CR1^I249/M280^*-expression in microglia-mediated inflammation in the diseased retina are relevant to identify mechanisms by which microglia contribute to disease progression. Our results show that *hCX3CR1^I249/M280^* mice are significantly more susceptible to microgliosis and production of *Cxcl10* and *TNFα* under acute inflammatory conditions. Inflammation is exacerbated under diabetic conditions and coincides with robust neuronal loss in comparison to *CX3CR1*-WT mice. Therefore, to further investigate the role of *hCX3CR1^I249/M280^*-expression in microglial responses, we pharmacologically depleted microglia using PLX-5622, a CSF-1R antagonist. PLX-5622 treatment led to a robust (~70%) reduction in Iba1^+^ microglia in all non-diabetic and diabetic mice. CSF-1R antagonism in diabetic *CX3CR1*-WT prevented TUJ1^+^ axonal loss, angiogenesis and fibrinogen deposition. In contrast, PLX-5622 microglia depletion in *CX3CR1*-KO and *hCX3CR1^I249/M280^* mice did not alleviate TUJ1^+^ axonal loss or angiogenesis. Interestingly, PLX-5622 treatment reduced fibrinogen deposition in *CX3CR1*-KO mice but not in *hCX3CR1^I249/M280^* mice, suggesting that *hCX3CR1^I249/M280^* expressing microglia influences vascular pathology differently compared to *CX3CR1*-KO microglia. Currently *CX3CR1*-KO mice are the most commonly used strain to investigate CX3CR1-FKN signaling effects on microglia-mediated inflammation and the results in this study indicate that *hCX3CR1^I249/M280^* receptor variants may serve as a complementary model to study dysregulated CX3CR1-FKN signaling. In summary, the protective effects of microglia depletion is *CX3CR1*-dependent as microglia depletion in *CX3CR1*-KO and *hCX3CR1^I249/M280^* mice did not alleviate retinal degeneration nor microglial morphological activation as observed in *CX3CR1*-WT mice.

## Introduction

Diabetic retinopathy (DR), an incurable eye disease caused by prolonged high glucose levels is a leading complication of diabetes mellitus and a common cause of blindness amongst working age adults (1). DR has been defined as a microvascular disease characterized by microaneurysms, intraretinal hemorrhaging, deposition of hard and soft exudates and abnormal angiogenesis (2). However, DR is also a neurodegenerative and neuroinflammatory disorder. Retinal ischemia leads to the development of cotton wool spots, stemming from the axoplasmic material of degenerating retinal ganglion cells (3). Microglia, the resident macrophages of the CNS, mediate a myriad of cellular functions in the retina, including synaptic pruning and wiring, surveillance and phagocytosis, and maintenance of neuroretinal homeostasis (4, 5). Microglia rapidly respond and become activated due to hyperglycemia and fluctuations in vascular vasoregulation (4, 6). More specifically, microglial contact with capillaries and pericytes is associated with reduced retinal blood flow in the early diabetic murine retina (6). In addition to inducing vascular abnormalities, microglia produce proinflammatory mediators including IL-1β, IL-6, TNF-α, vascular endothelial growth factor (VEGF) and reactive oxygen species (ROS) (7), and respond to circulating blood factors such as fibrin(ogen) (8–11). However, the exact mechanisms that initiate and regulate tissue damage in DR remain poorly understood.

CX3CR1, a receptor constitutively expressed by microglia in the CNS, and its ligand, fractalkine (FKN), a chemokine expressed uniquely in neurons, mitigates microglia mediated inflammation, often but not always tempering the severity of the reaction (12–16). A polymorphic variant of the *CX3CR1* gene encodes a protein with amino acid substitutions at residues 249 (valine substituted for isoleucine) and 280 (threonine substituted for methionine), *hCX3CR1^I249/M280^*, in ~25% of the population and produces an adhesive-defective receptor with decreased binding affinity for FKN (17, 18). In the diabetic murine retina, genetic and pharmacological inhibition of CX3CR1-FKN signaling inhibited diabetes associated vaso-constriction (6). In murine models of DR, absence of FKN or its receptor CX3CR1, lead to robust microglial activation, elevated release of pro-inflammatory mediators-IL-1β and TNF-α, and decreased production of anti-inflammatory cytokines IL-10 and IL-13, and enhanced vascular and neuronal damage (14, 19). Intravitreal delivery of recombinant soluble FKN decreased perivascular microglial clustering and retinal fibrinogen deposition in FKN-KO mice (19). In animal models of multiple sclerosis, mice expressing *hCX3CR1^I249/M280^* developed more severe clinical signs of experimental autoimmune encephalomyelitis (EAE) associated with increased cerebellar neuronal cell loss and demyelination (20). In the cuprizone-induced model of demyelination, *CX3CR1*-KO, *FKN*-KO, and *hCX3CR1^I249/M280^* expressing mice displayed delayed remyelination and decreased mature oligodendrocyte differentiation (21). Transcriptional analysis of *hCX3CR1^I249/M280^* expressing mice revealed enhanced gene expression patterns associated with inflammatory responses, production of reactive oxygen species and microglial activation under conditions of demyelination and neurodegeneration (20, 21). It is still uncertain how *CX3CR1*-variants affect disease development in DR.

Microglial depletion models are used to interrogate the role of microglia-mediated inflammation in disease initiation and progression (22–25). Utilizing the genetic model, CX3CR1^CreER^:R26^iDTR^, in which microglia become susceptible to the effects of diphtheria toxin (DTx), we recently showed that depletion and repopulation of microglia correlated with decreased neuronal cell loss and vascular damage in the diabetic retina (26). We also reported changes in the retinal transcriptome with reduced expression of complement-associated synaptic pruning and microglial activation genes (26). The CX3CR1-FKN signaling axis has also been shown to regulate microglial repopulation following ablation in CNS tissues, and delayed microglial repopulation is associated with lower *CX3CR1* expression (27, 28). Collectively these findings underscore the relevance of the CX3CR1-FKN signaling axis in regulating microglia-mediated inflammation.

To better understand the role of *CX3CR1*-variants in microglia-mediated inflammation, we utilized LPS-induced low-level endotoxemia and STZ-induced diabetes, as a two-hit model of diabetes and systemic inflammation. LPS was used to mimic the systemic pro-inflammatory environment common in diabetic patients as a result of persistent infections (29). This study aimed to determine how closely *hCX3CR1^I249/M280^* mice mirror the previously reported phenotype of *CX3CR1*-KO mice, showing enhanced microgliosis, and increased neuronal loss and vascular damage (14, 19). Moreover, we asked whether depletion of *CX3CR1*-WT, *CX3CR1*-KO or *hCX3CR1^I249/M280^* microglia *via* PLX-5622 confers the same retinal phenotype in diabetic mice. Our results show that low-level endotoxemia in *hCX3CR1^I249/M280^* mice increased microglial densities, angiogenesis and microglia perivascular clustering. Low-level endotoxemia significantly increased the expression of *Cxcl10* and *TNF-α* in *hCX3CR1^I249/M280^* mice in comparison to *CX3CR1*-WT mice. Diabetic *CX3CR1*-KO and *hCX3CR1^I249/M280^* retinas revealed decreased TUJ1^+^ axonal immunoreactivity in comparison to *CX3CR1*-WT mice. PLX-5622 treatment led to ~70% reduction in Iba1^+^ microglia in all non-diabetic and diabetic groups. PLX-5622 microglia depletion in *CX3CR1*-WT mice correlated with prevention of TUJ1^+^ axonal loss, and ameliorated vascular damage and fibrinogen deposition in the diabetic retina. However, PLX-5622 treatment in *CX3CR1*-KO and *hCX3CR1^I249/M280^* mice did not prevent TUJ1^+^ axonal loss, nor abnormal angiogenesis in the diabetic retina. Together these results highlight that the neuro- and vasculo-protective effects of microglia depletion in the diabetic retina is *CX3CR1*-dependent.

## Materials and methods

### Mice

All experiments used male mice as female mice do not develop consistent hyperglycemic levels in response to STZ due to the antidiabetic actions elicited by 17β-estradiol (14, 30, 31). Male mice were 6-8 weeks of age at the time of STZ-induced diabetes, as STZ treatment in older rodents has been shown to lead to a high mortality rate (32). CXC3R1-WT (JAX stock number: 000664; RRID : IMSR_JAX:000664) and CX3CR1-KO (JAX stock number: 005582; RRID : IMSR_JAX:005582) mice were purchased from The Jackson Laboratory. Mice expressing the human CX3CR1 variant (hCX3CR1^I249/M280^) were bred and maintained as previously described (20). Mice containing the human CX3CR1 I250 or M280 polymorphism within the mouse CX3CR1 loci, mCX3CR1^I250^ and mCX3CR1^M281^ respectively, were obtained from Biogen ^©^. Mice were maintained at the Laboratory Animal Resource Center at The University of Texas at San Antonio under conventional housing conditions. All experiments were performed in accordance with National Institutes of Health guidelines and approved by UTSA-Institutional Animal Care and Use Committee.

### Two hit model of streptozotocin-induced hyperglycemia and LPS-induced systemic inflammation

**Figure 1:**
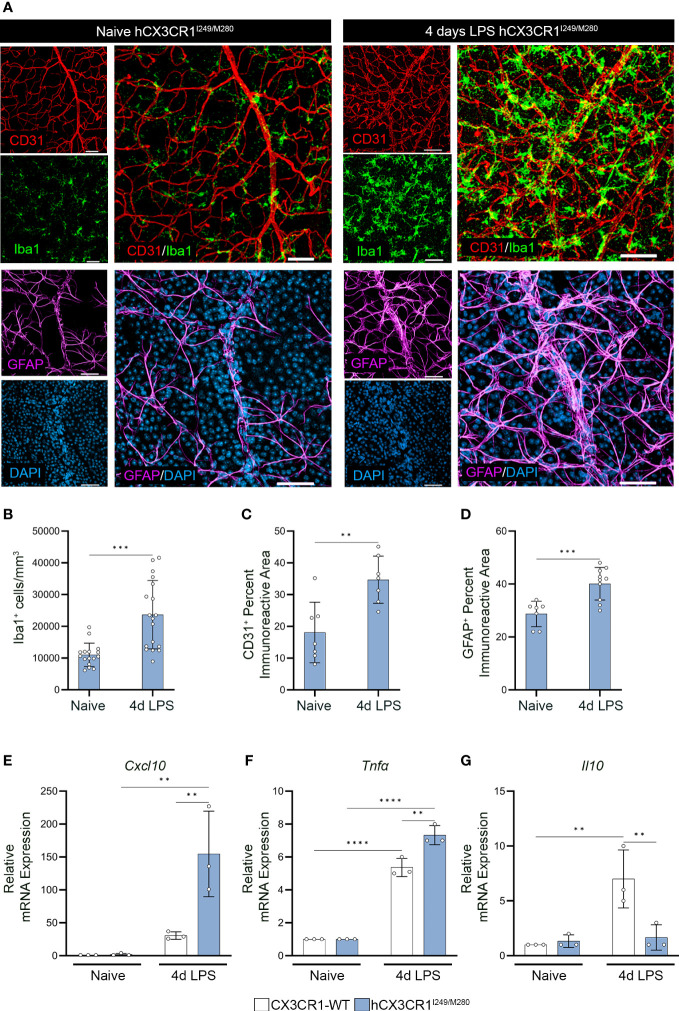
Acute LPS-induced inflammation induces aberrant angiogenesis and gliosis in the *hCX3CR1^I249/M280^* retina. *hCX3CR1^I249/M280^* mice were i.p. injected with 0.08mg/Kg LPS once daily for four days (4d), and naïve age-matched controls received PBS. **(A)** Confocal images of retinal tissues stained for Iba1 (green), CD31 (Red), GFAP (magenta) and DAPI (blue) in naïve and 4d LPS *hCX3CR1^I249/M280^* mice. Confocal images represent the retinal ganglion cell layer in the peripheral retina. **(B–D)**, Quantification of retinal IHC analysis for Iba1^+^ cells/mm^3^ **(B)**, CD31^+^ percent immunoreactive area **(C)**, and percent immunoreactive area for GFAP **(D)**. **(E–G)**, Graphical representation for RT-qPCR analysis of retinas for relative mRNA expression for *Cxcl10* **(E)**, *Tnfα* **(F)** and *Il10* **(G)**. Data show the average of the 2 central, 2 medial and 2 peripheral images taken per mouse. Data show mean ± SD, *n* = 3 to 10 mice per group where each dot represents an individual mouse. **P<0.01, ***P<0.001, ****P<0.0001 using Student’s t-test, with Welch’s correction. Scale bars measure 50µm.

**Figure 2:**
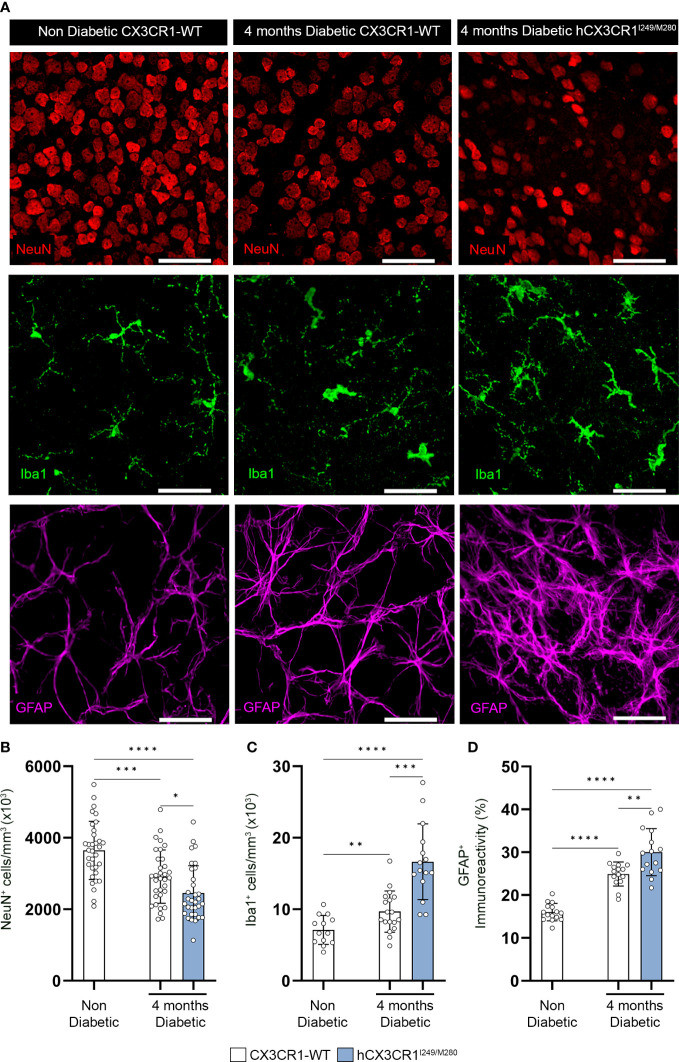
*hCX3CR1^I249/M280^* variant is associated with increased neuronal cell loss and reactive gliosis in the diabetic retina. Hyperglycemia was induced in *CX3CR1*-WT and *hCX3CR1^I249/M280^* mice *via* i.p. injection with STZ and retinal tissues were analyzed after four months of hyperglycemia. **(A)** Confocal images of retinal tissues stained for Iba1 (green), NeuN (Red) and GFAP (magenta) in naïve *CX3CR1*-WT and 4-months diabetic *CX3CR1*-WT and *hCX3CR1^I249/M280^* mice. Confocal images represent the retinal ganglion cell layer in the peripheral retina. B-D, Quantification of retinal IHC analysis for NeuN^+^ cells/mm^3^ **(B)**, Iba1^+^ cells/mm^3^ **(C)**, and percent immunoreactive area for GFAP **(D)**. Data show the 2 central, 2 medial and 2 peripheral images taken per mouse. Data show mean ± SD, *n* = 4 to 10 mice per group where each dot represents an individual mouse. *P<0.05, **P<0.01, ***P<0.001 ****P<0.0001 using Student’s *t*-test, with Welch’s correction. Scale bars measure 50µm.

**Figure 3:**
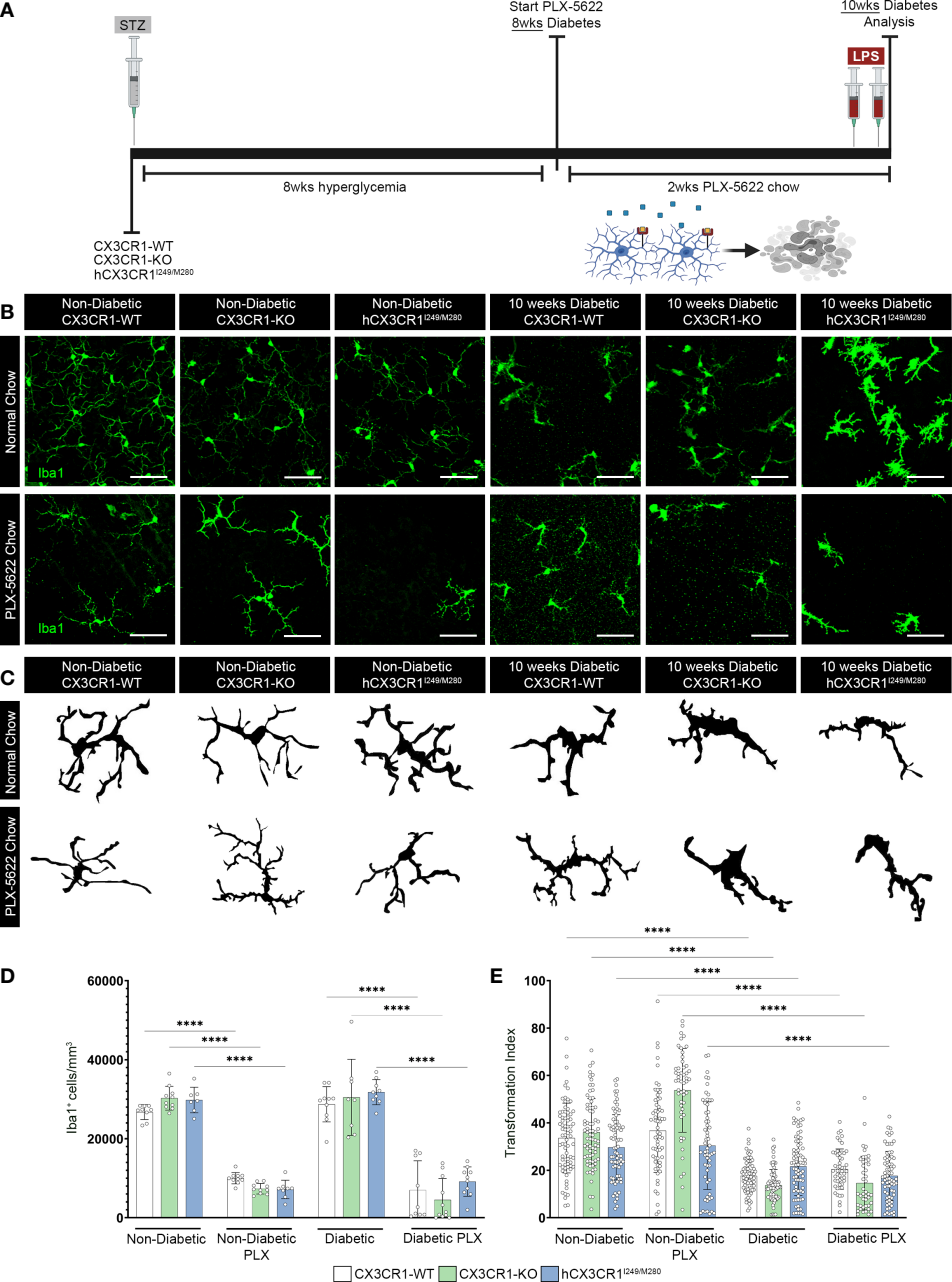
CSF-1R antagonism induces robust microglia depletion in the diabetic retina. **(A)** Microglia were pharmacologically depleted using PLX-5622 in non-diabetic and 8-wks diabetic *CX3CR1*-WT, *CX3CR1*-KO, and *hCX3CR1^I249/M280^* mice for two weeks. Non-diabetic control mice received citrate buffer. Non-depleted, non-diabetic and diabetic controls remained on normal chow. Confocal images of retinal tissues stained for Iba1 (green) **(B)** and transformation index cellular tracings **(C)** in *CX3CR1*-WT, *CX3CR1*-KO and *hCX3CR1^I249/M280^* mice. Confocal images represent the retinal ganglion cell layer in the peripheral retina. **(D, E)**, Quantification of retinal IHC analysis for Iba1^+^ cells/mm^3^ **(D)** and transformation index **(E)**. Data show the average of the 2 central, 2 medial and 2 peripheral images taken per mouse. Data show mean ± SD, *n* = 6 to 10 mice per group where each dot represents an individual mouse **(D)**. Transformation index data show mean ± SD *n* = 44 to 75 microglia per group for *n*=5 mice where each dot represents an individual microglia cell **(E)**. ****P<0.0001 using 2-way ANOVA. Scale bars measure 50µm.

**Figure 4:**
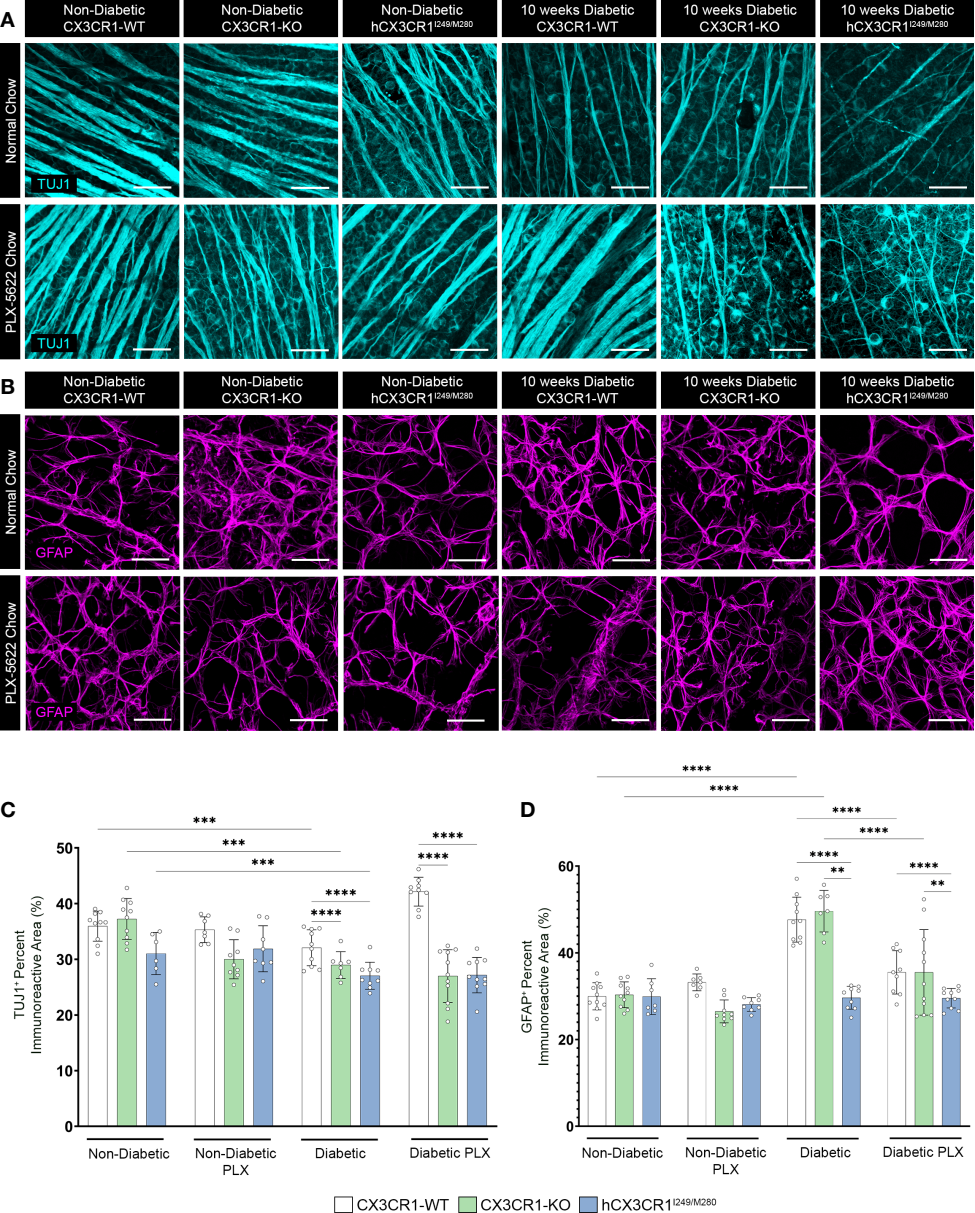
PLX-5622 treatment does not prevent TUJ1^+^ axonal loss in the diabetic *CX3CR1*-KO and *hCX3CR1^I249/M280^* retinas. Microglia were pharmacologically depleted using PLX-5622 in non-diabetic and 8-wks diabetic *CX3CR1*-WT, *CX3CR1*-KO, and *hCX3CR1^I249/M280^* mice for two weeks. Non-diabetic control mice received citrate buffer. Non-depleted, non-diabetic and diabetic controls remained on normal chow. Confocal images of retinal tissues stained for TUJ1 (turquoise) **(A)** and GFAP (magenta) **(B)** in *CX3CR1*-WT, *CX3CR1*-KO and *hCX3CR1^I249/M280^* mice. Confocal images represent the retinal ganglion cell layer in the peripheral retina. **(C, D)**, Quantification of retinal IHC analysis for TUJ1^+^ percent immunoreactive area **(C)** and GFAP^+^ percent immunoreactive area **(D)**. Data show the average of the 2 central, 2 medial and 2 peripheral images taken per mouse. Data show mean ± SD, *n* = 6 to 10 mice per group where each dot represents an individual mouse. **P<0.01, ***P<0.001, ****P<0.0001 using 2-way ANOVA. Scale bars measure 50µm.

**Figure 5:**
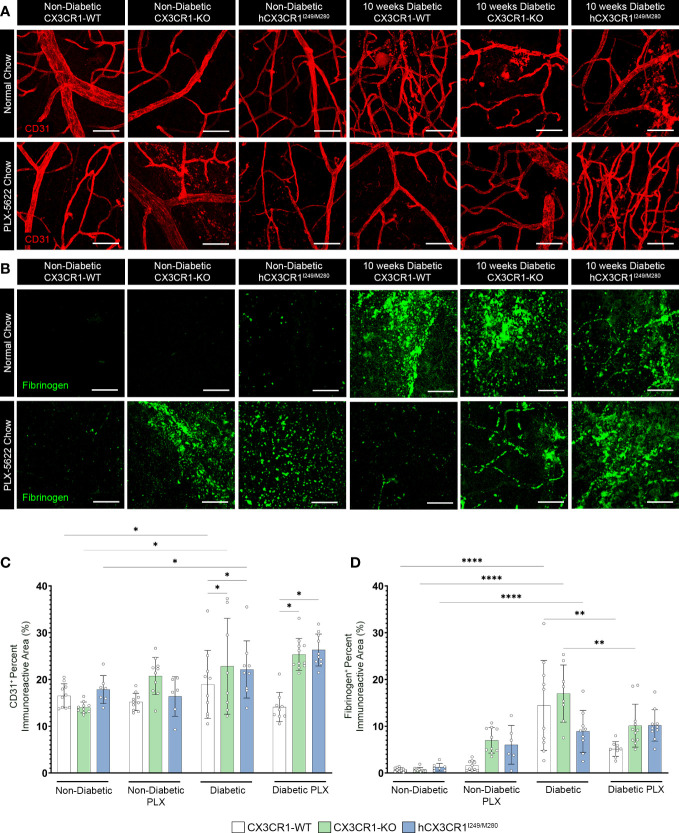
PLX-5622 treatment does not alleviate angiogenesis in the diabetic *CX3CR1*-KO and *hCX3CR1^I249/M280^* retina. Microglia were pharmacologically depleted using PLX-5622 in non-diabetic and 8-wks diabetic *CX3CR1*-WT, *CX3CR1*-KO, and *hCX3CR1^I249/M280^* mice for two weeks. Non-diabetic control mice received citrate buffer. Non-depleted, non-diabetic and diabetic controls remained on normal chow. Confocal images of retinal tissues stained for CD31 (red) **(A)** and fibrinogen (green) **(B)** in *CX3CR1*-WT, *CX3CR1*-KO and *hCX3CR1^I249/M280^* mice. Confocal images represent the retinal ganglion cell layer in the peripheral retina. **(C, D)**, Quantification of retinal IHC analysis for CD31^+^ percent immunoreactive area **(C)** and fibrinogen^+^ percent immunoreactive area **(D)**. Data show the average of the 2 central, 2 medial and 2 peripheral images taken per mouse. Data show mean ± SD, *n* = 6 to 10 mice per group where each dot represents an individual mouse. *P<0.05, **P<0.01, ****P<0.0001 using 2-way ANOVA. Scale bars measure 50µm.

Mice were intra-peritoneally (i.p.) injected once daily for five days with 60 mg/Kg of streptozotocin (STZ) to induce hyperglycemia (Sigma Aldrich catalog number: S0130) (19, 33). Age-matched non-diabetic controls received citrate buffer as a vehicle control. Animals were deemed hyperglycemic when blood glucose levels were > 250 mg/dL and blood glucose levels were measured weekly. Diabetic patients experience recurrent infections, low-grade systemic inflammation and increased plasma levels of lipopolysaccharide (LPS) (12, 34–36). Therefore, to mirror these manifestations, mice were treated with 0.08mg/kg LPS. Non-diabetic mice (**Figure 1** and **Supplementary Figure 1**) received one daily injection of 0.08mg/kg LPS for four consecutive days. Due to these mice lacking the systemic inflammation caused by diabetes, we challenged them with 4 days of LPS to induce low level endotoxemia as previously characterized (19). Four months diabetic mice (**Figure 2**) did not receive LPS treatment, due to their increased sensitivity to cachexia at this more chronic stage of diabetes. Acute LPS treatment can cause hypoglycemia and induce large variation in circulating glucose levels in diabetic mice (37, 38). Therefore, to avoid large fluctuations in glucose levels and to prevent masking hyperglycemia, 10 weeks diabetic mice, (**Figures 3**–**5** and **Supplementary Figures 2**–**6**) received one daily injection of 0.08mg/kg LPS for 2 consecutive days prior to euthanasia as previously characterized (26).

### Pharmacological PLX-5622 microglia depletion

Eight-wks following STZ-induced hyperglycemia, CX3CR1-WT, CX3CR1-KO, hCX3CR1^I249/M280^, mCX3CR1^I250^ and mCX3CR1^M281^ mice were fed 0.12% PLX-5622 (MedChemExpress, catalog number: HY-114153) chow (7012, Blue, TD.200435, Envigo) for 2-wks, until 10-wks of hyperglycemia, the time point of tissue collection.

### Tissue collection

Mice were transcardially perfused with cold 1x Hanks’s Balanced Salt Solution (HBSS). Eyes were enucleated and placed in 4% paraformaldehyde (PFA) for 20 minutes. Next, retinas were dissected out of the globe of the eye and placed in 1% PFA for 1 hour. Fixed retinas were then place in cryoprotection solution (200 mL glycerol, 200 mL 0.4M Sorenson’s buffer and 600 mL MilliQ water) overnight at 4°C, and the following day placed in cryostorage solution (500 mL 0.2M PO_4_, 10 g PVP-40, 300 g sucrose and 300 mL ethylene glycol) at -20°C. Brain tissues were dissected from the skull and fixed in 4% PFA overnight at 4°C followed by cryoprotection overnight at 4°C. Cryoprotected brains were sectioned at 30µm using a freezing microtome and free floating brain sections were placed in cryostorage solution at -20°C.

### Immunofluorescent staining

Using the optic disc to center the retina, whole retinas were divided into 4 leaflets that each contained the central, medial and peripheral retina (**Supplementary Figure 1A**). ¼ leaflets were chosen at random to visualize proteins of interest. Two images were obtained per region for a total of 6 images per ¼ retinal leaflet (**Supplementary Figure 1A**). Retinal leaflet preparations were blocked overnight in 10% goat serum containing 1% Triton-X 100 at 4°C for immunohistochemical analysis. Tissues were then incubated overnight at 4°C with primary antibodies diluted in blocking solution (10% goat or donkey serum containing 1% Triton-X 100) to visualize proteins of interest, rabbit anti-ionized calcium binding adaptor molecule-1 (Iba1) (RRID: AB_839504), mouse anti-neuronal nuclei (NeuN) (RRID: AB_2298772), mouse anti-β tubulin III (TUJ1) (RRID: AB_10063408), rat anti-glial fibrillary acidic protein (GFAP) (RRID: AB_2532994), rat anti-pecam-1 (CD31) (RRID: AB_393571), and rabbit anti-fibrinogen (RRID: AB_2894406). To remove unbound antibodies, tissues underwent 7 washes each for 5 minutes in PBS/0.1% Triton-X 100. Tissues were incubated for three hours in species-specific secondary antibodies to visualize proteins of interest followed by 7 washes each at 5 minutes in PBS with 0.1% Triton-X 100. To label cellular nuclei, tissues were incubated in Hoechst 3342 (Thermo Fisher Scientific catalog number: H1399) for 7 minutes, followed by 3 washes in PBS for 5 minutes each. Tissues were mounted on Superfrost Plus microscope slides (Fisher Scientific catalog number: 12-550-15) and cover slipped using Fluorsave (Millipore Sigma catalog number: 345789). Antibody combinations and species-specific secondary antibody combinations are outlined in **Supplementary Table 1**.

### Confocal microscopy and image analysis

Confocal microscopy was performed using a Zeiss 710 NLO confocal microscope and 3D compositions of confocal images were generated using Imaris software v7.2 (Bitplane). Six images were obtained per ¼ retinal leaflet, 2 images at the central retina nearest the optic nerve, 2 images in the middle of the leaflet and 2 images in the outer leaflet, per mouse (**Supplementary Figure 1A**). Quantifications shown represent the average of the six images taken per mouse. To quantify Iba1^+^ microglial and NeuN^+^RBPMS^+^ neuronal cell body densities, cells were manually counted in 40x images using the counter tool in Adobe Photoshop version 21.0.3. To quantify the percent immunoreactive area of TUJ1^+^ axons, GFAP^+^ glia, CD31^+^ blood vessels and fibrinogen, raw confocal images were converted to 32-bit in ImageJ Fiji analysis software (NIH) and an automatic threshold was applied. Data was normalized by volume based on X, Y and Z coordinates (i.e. 212µm *212 µm *Z stack thickness) to account for changes in confocal Z-stack thickness and images size (scale settings- distance in pixels: 1024; known distance: 206.25). We analyzed microglial morphological changes by determining the transformation index (TI) of microglia. To measure TI, 40x images were converted to 32-bit in ImageJ Fiji analysis software (NIH) and individual microglial cells were traced to determine the perimeter and area of a microglia cell. TI was calculated using the equation: perimeter^2^/4π × area^2^ (39). The TI was determined for 5 microglia per 40x image, spanning the 3 regions of the retina, central, medial and peripheral retina from 5 mice per treatment and genotype for a total of 15 microglia quantified per animal. Values are expressed as a range from 1 to 100, with a TI value closer to 1 representing a circular, amoeboid microglial cell with fewer and/or shorter cellular processes. Higher TI values represent ramified microglial cells with extensive branching and smaller cell bodies.

### RNA isolation and RT-qPCR analysis

Retinal isolates from enucleated retinas were homogenized in Trizol (Invitrogen catalog number: 15-596-018) followed by RNA isolation using the Qiagen RNeasy Kit (Qiagen catalog number: 74104). To generate cDNA, 500 ng of total RNA was reverse-transcribed using the High-capacity cDNA Reverser Transcription Kit (Thermo Fisher Scientific catalog number: 4368814). The 7900 HT Fast Real-Time PCR system was used to run RT-qPCR reactions were ran in a 384-well plate in triplicates using SYBR Green PCR master mix (Thermo Fisher Scientific catalog number: 4344463), 250 nM forward and reverse primers and 20ng of cDNA template. Results were analyzed as previously described using the comparative Ct method (14, 21). Data was normalized to two housekeeping genes, *Rn18s* (18s) and *Actb* (β-actin) and is presented as the fold change relative to genotype-specific naïve controls. The following primers were used: *18s* (Accession number: NR_003278.3): CGGCTACCACATCCAAGGAA (forward),

GTCGGAAATACCGCGGTC (reverse); *β-actin* (Accession number: NM_007393) 5’-CTCTGGCTCCTAGCACCATGAAGA-3’ (forward), 5’-GTAAAACGCAGCTCAGTAACAGTCCG-3’ (reverse); *Cxcl10* (Accession number: NM_021274.2): 5’-TGCTGCCGTCATTTTCTG-3’ (forward), 5’-GCTCGCAGGGATGATTTCAAG-3’ (reverse); *Il1β* (Accession number: NM_008361.3): 5’-GTGTGGATCCAAAGCAATAC-3’ (forward), 5’-GTCTGCTCATTCATGACAAG-3’ (reverse); *Nos2* (Accession number:NM_010927.4): 5’-GGCAGCCTGTGAGACCTTTG-3’ (forward), 5’-TGCATTGGAAGTGAAGCGTTT-3’ (reverse); *Tnfα* (Accession number: NM_013639.3): 5’- GGTGCCTATGTCTCAGCCTCTT-3’ (forward), 5’- GCCATAGAACTGATGAGAGGGAG-3’ (Reverse); *Il6* (Accession number: NM_031168.1): 5’- TACCACTTCACAAGTCGGAGGC-3’ (forward), 5’- CTGCAAGTGCATCATCGTTGTTC-3’ (reverse); *Il10* (Accession number: NM_010548.1): 5’- CGGGAAGACAATAACTGCACCC-3’ (forward), 5’- CGGTTAGCAGTATGTTGTCCAGC-3’ (reverse).

### Statistical analyses

All analyses were conducted using GraphPad Prism v9.2 and a *P* value <0.05 was considered statistically significant. Statistical significance is denoted as *P value <0.05, **P value <0.01, ***P value <0.001 and ****P value <0.0001. Statistical tests performed included a two-tailed parametric unpaired student’s *t* test with Welch’s correction when comparing two groups (**Figures 1**, **2** and **Supplementary Figure 3E**). When comparing multiple groups, a two-way ANOVA with the Tukey’s *post-hoc* test was performed, using the treatment type as the first variable and genotype as the second variable (**Figures 3**–**5** and **Supplementary Figures 3D**, **4**, **5**).

## Results

### Low-level systemic endotoxemia induces robust inflammation in the *hCX3CR1^I249/M280^* retina

To visualize changes to the vasculature and glial responses, retinal tissues were stained with Iba1 (microglia), CD31 (endothelial cells) and GFAP (astrocytes) (**Figure 1**). Four-day LPS treatment induced a significant increase in Iba1^+^ cells (23657 ± 10762, student’s *t* test *P*=0.0001) in comparison to naïve controls (11028 ± 3657) (**Figures 1A, B**). Additionally, LPS treatment increased CD31^+^ percent immunoreactive area (34.69 ± 7.423, student’s *t* test *P*=0.0037) and GFAP^+^ astrogliosis (40.13 ± 6.143, students *t* test *P*=0.0007) in comparison to PBS naive controls (CD31^+^: 18.09 ± 9.503; GFAP^+^: 28.7 ± 4.811) (**Figures 1A, C, D**). Microglial clustering around the vasculature was evident in LPS treated *hCX3CR1^I249/M280^* mice in contrast to naïve *hCX3CR1^I249/M280^* mice (**Figure 1A**). RT-qPCR analysis of retinal RNA isolated from LPS treated *hCX3CR1^I249/M280^* mice revealed a significant increase in gene expression for proinflammatory cytokines *Cxcl10* and *Tnfα*, and a decrease in the anti-inflammatory cytokine *Il10* (**Figures 1E–G**). Diabetes induced a significant increase in gene expression for *Il1β* and *Il6* compared to ND mice, regardless of genotype (**Supplementary Figures 1B, C**). These data suggest that *hCX3CR1^I249/M280^* mice are more susceptible to a proinflammatory response under acute inflammatory conditions.

### Retinal pathology in diabetic *hCX3CR1^I249/M280^* mice is associated with increased neuronal cell loss and reactive gliosis

Immunohistochemical analysis of retinal tissues after four months of hyperglycemia revealed a decrease in NeuN^+^ neuronal densities in diabetic *CX3CR1*-WT and *hCX3CR1^I249/M280^* mice in comparison to the non-diabetic *CX3CR1*-WT (3646 ± 811.7) control group (**Figures 2A, B**). Notably, diabetic *hCX3CR1^I249/M280^* mice showed a statistically significant decrease in NeuN^+^ neuronal densities (2451 ± 766.8, student’s t test, P<0.0001) compared to diabetic *CX3CR1*-WT mice (2911 ± 745.3, student’s *t* test, *P*=0.0003) (**Figures 2A, B**). *hCX3CR1^I249/M280^* mice also showed an increase in Iba1^+^ cells/mm^3^ (16.64 x10^3^ ± 5.289, student’s *t* test, *P*=0.0002) and GFAP^+^ percent immunoreactive area (30.01 ± 5.513, student’s *t* test, *P*=0.0044) compared to diabetic *CX3CR1*-WT mice (Iba1: 9.679 x10^3^ ± 2.879; GFAP: 24.91 ± 2.797) (**Figures 2A, C, D**).

### PLX-5622 treatment induces robust microglia depletion in the diabetic retina

To further investigate the role of aberrantly activated microglia, in the absence of *CX3CR1* (*CX3CR1*-KO) or expression of the human variant alleles of *CX3CR1* (*hCX3CR1^I249/M280^*), in the diabetic retina, mice were treated for 2-wks with PLX-5622 (**Figure 3A**). To visualize genotype-specific changes in the degree of microglia depletion targeted with PLX-5622 treatment and microglial reactivity, we quantified the number of Iba1^+^ cells and transformation index (TI), respectively, in PLX-5622 treated and normal chow mice (**Figure 3**). PLX-5622 treatment resulted in a significant reduction in Iba1^+^ microglia in ND mice, *CX3CR1*-WT (10015.542 ± 1422.395, 2-way ANOVA *P*<0.0001), *CX3CR1*-KO (7342.444 ± 1326.141, 2-way ANOVA *P*< 0.0001) and *hCX3CR1^I249/M280^* (7146.941 ± 2337.299, 2-way ANOVA *P*<0.0001) in comparison to their respective ND genotype-matched controls, *CX3CR1*-WT (26733.516± 1888.129), *CX3CR1*-KO (30248.529 ± 3004.598) and *hCX3CR1^I249/M280^* (29796.14 ± 3240.632) (**Figures 3B, D**). Consistent with these results, under diabetic conditions PLX-5622 treatment led to a significant reduction in the number of Iba1^+^ cells in the retina and there were no differences between the degree of depletion across the *CX3CR1*-WT (6994.483 ± 7421.66), *CX3CR1*-KO (4537.517 ± 5401.353) and *hCX3CR1^I249/M280^* (9155.333 ± 3713.293) diabetic groups (**Figures 3B, D**). Overall, PLX-5622 treatment led to a robust ~70% reduction in Iba1^+^ microglia in all non-diabetic and diabetic mice. Microglia retained high TI values with a ramified morphology and small cell bodies in *CX3CR1*-WT (36.772 ± 17.621), *CX3CR1*-KO (53.73 ± 17.67) and *hCX3CR1^I249/M280^* (30.398 ± 18.472) in PLX-treated ND mice, compared to ND normal chow *CX3CR1*-WT (33.607 ± 14.709) *CX3CR1*-KO (36.01 ± 14.22) and *hCX3CR1^I249/M280^* (29.711 ± 13.76) controls (**Figures 3B, E**). Retinal Iba1^+^ cells displayed a significant reduction in TI values in all diabetic groups, with microglia displaying an ameboid morphology with retracted cellular processes and large cell bodies (**Figures 3C, E**).

### CSF-1R antagonism prevents TUJ1^+^ axonal loss in the diabetic *CX3CR1*-WT retina but not in the diabetic *CX3CR1*-KO or *hCX3CR1* ^I249/M280^ retina

We next assessed the effects of PLX-5622 treatment on TUJ1^+^ and GFAP^+^ percent immunoreactive areas (**Figure 4**). Analysis of TUJ1^+^ retinal axons revealed that PLX treatment in ND mice did not alter TUJ1^+^ percent immunoreactive area in comparison to ND normal chow controls (**Figures 4A, C**). Diabetes led to a robust decrease in TUJ1^+^ percent immunoreactive area in all genotypes when compared to ND controls (**Figures 4A, C**). However, diabetic *CX3CR1*-KO (28.969 ± 2.393, 2-way ANOVA *P*<0.0001) and *hCX3CR1^I249/M280^* (27.047 ± 2.43, 2-way ANOVA *P*<0.0001) mice were significantly more susceptible to TUJ1^+^ axonal loss in comparison to diabetic *CX3CR1*-WT (32.078 ± 3.225) mice (**Figures 4A, C**). TUJ1^+^ axonal loss was prevented in PLX-5622 treated diabetic *CX3CR1*-WT mice (42.155 ± 2.59) in comparison to the diabetic *CX3CR1*-WT normal chow (32.078 ± 3.25) control, closely mirroring the levels of TUJ1^+^ percent immunoreactivity found in the ND normal chow control group (35.953 ± 2.703) (**Figures 4A, C**). In contrast, PLX-5622 treatment in diabetic *CX3CR1*-KO and *hCX3CR1* ^I249/M280^ mice did not alleviate TUJ1^+^ axonal loss caused by diabetes (**Figures 4A, C**). When we assessed GFAP^+^ glial cell responses to PLX-5622 treatment in ND mice, there were no observable changes in GFAP^+^ percent immunoreactive area in comparison to ND normal chow controls (**Figures 4B, D**). Diabetes induced a significant increase in GFAP^+^ percent immunoreactive area in *CX3CR1*-WT (47.64 ± 5.179, 2-way ANOVA *P*<0.0001) and *CX3CR1*-KO (49.60 ± 4.794, 2-way ANOVA *P*<0.0001) mice in comparison to ND controls, (*CX3CR1*-WT 29.969 ± 3.127 and *CX3CR1*-KO 30.325 ± 2.976; **Figures 4B, D**). PLX-5622 treatment led to a reduction in GFAP^+^ percent immunoreactive area in diabetic *CX3CR1*-WT (35.49 ± 4.996, 2-way ANOVA *P*<0.0001) and *CX3CR1*-KO (35.49 ± 9.865, 2-away ANOVA *P*=0.0001) in comparison to their diabetic *CX3CR1*-WT (47.639 ± 5.179) and *CX3CR1*-KO (49.601 ± 4.794) controls, respectively, closely resembling non-diabetic normal chow, *CX3CR1*-WT (29.969 ± 3.127) and *CX3CR1*-KO (30.325 ± 2.976) mice, respectively (**Figures 4B, D**).

### CSF-1R antagonism does not alleviate aberrant angiogenesis in the diabetic CX3CR1-deficient retina

We assessed vascular abnormalities in the diabetic retina and measured fibrinogen deposition and extravasation from the vasculature (**Figure 5**). PLX-5622 treatment in ND mice did not alter CD31^+^ percent immunoreactive area in *CX3CR1*-WT (15.209 ± 1.863), *CX3CR1*-KO (20.751 ± 3.962) and *hCX3CR1^I249/M280^* (16.373 ± 4.219) mice compared to ND normal chow *CX3CR1*-WT (16.51 ± 2.565), *CX3CR1*-KO (14.017 ± 1.168) and *hCX3CR1^I249/M280^* (17.842 ± 2.977) controls, respectively (**Figures 5A, C**). Diabetes induced a significant increase in CD31^+^ percent immunoreactive area in all genotypes when compared to ND controls (**Figures 5A, C**). However, *CX3CR1*-KO (22.82 ± 10.256, 2-way ANOVA *P*=0.0143) and *hCX3CR1* ^I249/M280^ (22.145 ± 6.108, 2-way ANOVA *P*=0.0156) mice were more susceptible to an increase in CD31^+^ percent immunoreactive area indicative of more angiogenesis and vascular damage (**Figures 5A, C**). In diabetic mice ruptured and discontinuous blood vessels with aggregated endothelium were observed (**Figure 5A**). This phenotype was ameliorated in diabetic PLX-5622 treated *CX3CR1*-WT mice (14.098 ± 3.115), with a ~25% reduction in CD31^+^ percent immunoreactive area compared to diabetic normal chow *CX3CR1*-WT mice (18.926 ± 7.27), closely resembling their ND, normal chow *CX3CR1*-WT (16.51 ± 2.565) controls (**Figures 5A, C**). However, PLX-5622 treatment did not alleviate CD31^+^ angiogenesis nor abnormal vascular pathology in the diabetic *CX3CR1*-KO (25.348 ± 3.418) and *hCX3CR1^I249/M280^* (26.305 ± 3.417) retina, compared to ND *CX3CR1*-KO (16.51 ± 2.565) and *hCX3CR1^I249/M280^* (17.842 ± 2.977) controls, respectively (**Figures 5A, C**). PLX-treated diabetic *CX3CR1*-KO (25.348 ± 3.418, 2-way ANOVA *P*=0.0143) and *hCX3CR1^I249/M280^* (26.305 ± 3.417, 2-way ANOVA *P*=0.0156) mice had significantly higher CD31^+^ percent immunoreactive areas in comparison to *CX3CR1*-WT (14.098 ± 3.115) mice (**Figures 5A, C**). Fibrinogen deposition was comparable in ND and ND PLX-5622 treated groups (**Figures 5B, D**). Diabetes led to an increase in fibrinogen deposition in the diabetic, normal chow retina in all genotypes (**Figures 5B, D**). Fibrinogen deposits were visualized as aggregates near areas of ruptured endothelium (**Figure 5B**). PLX-5622 treatment led to a significant ~64% reduction in fibrinogen deposition in diabetic *CX3CR1*-WT (5.141 ± 1.563, 2-way ANOVA *P*=0.012) mice and a ~40% reduction in *CX3CR1*-KO (10.12 ± 4.575, 2-way ANOVA *P*=0.012) mice with respect to their diabetic, normal chow, *CX3CR1*-WT (14.42 ± 9.626) and *CX3CR1*-KO (16.998 ± 6.096) controls, respectively (**Figures 5B, D**). Contrary to *CX3CR1*-WT and *CX3CR1*-KO mice, PLX-5622 treatment in the diabetic *hCX3CR1^I249/M280^* retina (10.198 ± 3.355) did not significantly alter the percent of fibrinogen deposition in comparison to their diabetic, normal chow *hCX3CR1^I249/M280^* (8.913 ± 4.469) control (**Figures 5B, D**).

## Discussion

**Figure 6:**
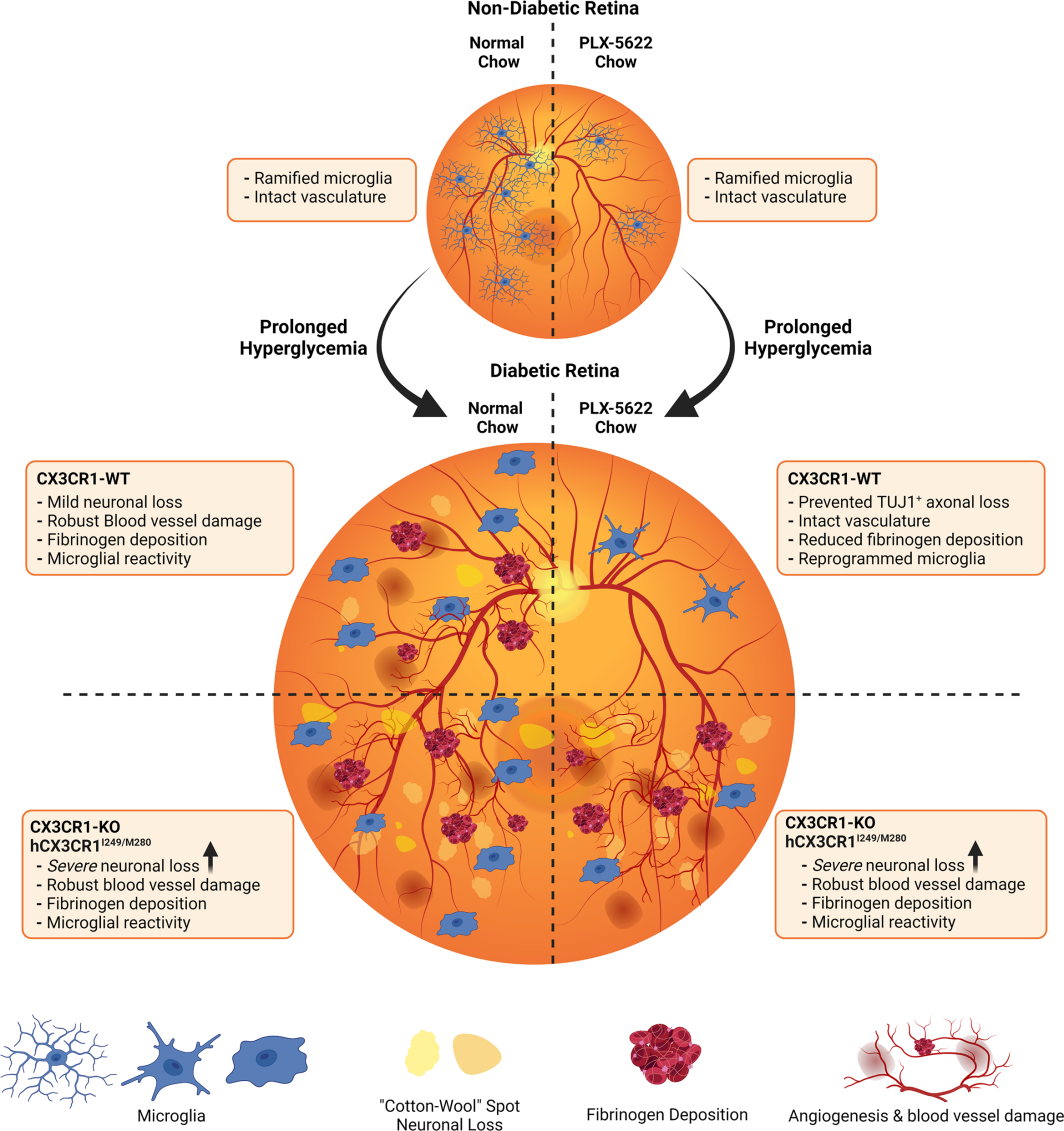
Schematic summary: Pharmacological depletion of microglia alleviates neuronal and vascular damage in the diabetic *CX3CR1*-WT retina but not in *CX3CR1*-KO or *hCX3CR1^I249/M280^*-expressing retina. In the non-diabetic retina, normal chow and PLX-5622 chow treated *CX3CR1*-WT, *CX3CR1*-KO, and *hCX3CR1^I249/M280^* mice have intact vasculature and ramified microglia. PLX-5622 chow treatment induces robust microglia depletion in *CX3CR1*-WT, *CX3CR1*-KO, and *hCX3CR1^I249/M280^* mice. Prolonged hyperglycemia results in angiogenesis, vascular damage and fibrinogen deposition in *CX3CR1*-WT, *CX3CR1*-KO, and *hCX3CR1^I249/M280^* mice. Additionally, diabetic *CX3CR1*-KO, and *hCX3CR1^I249/M280^* mice are more susceptible to neuronal and axonal loss. CSF-1R antagonism alleviates vascular damage and fibrinogen deposition and prevents neuronal loss in the diabetic *CX3CR1*-WT retina. In contrast, CSF-1R antagonism in *CX3CR1*-KO, and *hCX3CR1^I249/M280^* mice does not prevent vascular damage, fibrinogen deposition and neuronal loss.

Microglia support tissue homeostasis to include, synaptic pruning and rewiring *via* complement mediated deposition, the secretion of neurotrophic factors to promote in the survival and maintenance of neurons, immune surveillance and phagocytosis, vascular remodeling and the secretion of factors to support myelinogenesis and oligodendrocyte progenitor cell (OPC) maintenance. As notably plastic cells, the various roles microglia perform are largely governed by their activation state (40, 41). Under steady-state conditions, microglia maintain a ramified morphology, with small cell bodies and long cellular processes that they use to extend and retract to survey the CNS (42). Under diseased conditions or when microglia receive environmental cues of stress, microglia will acquire an amoeboid morphology more consistent with the morphology of phagocytic macrophages (42). These two morphologies of microglial activation initially described as “M1”, proinflammatory microglia, or “M2”, anti-inflammatory microglia (43) do not recapitulate their range of functions and it is now understood that microglia responses involve a spectrum of phenotypic and ‘omics’ changes (transcriptomics, proteomics, or metabolomics). Of the many variables regulating microglia inflammatory states, CX3CR1-FKN signaling has been shown to be heavily involved in regulating microglial responses in the retina to include vasoregulation, vascular damage, neuronal cell loss and proinflammatory cytokine production (6, 14, 19). Here we show that microglial depletion mediated by PLX-5622 is not 100% effective in the retina and that decreased microglial densities appear neuroprotective in *CX3CR1*-WT retinas but not in *CX3CR1*-KO or *hCX3CR1^I249/M280^* mice (**Figure 6**). However, based on other studies it is likely that the microglia population detected after depletion arises from a depletion-resistant pool with phenotypic features that resembled their ancestor.

*CX3CR1* deficiency has been shown to lead to a robust increase in proinflammatory gene expression including *Il1β* and *Nos2* in models of systemic endotoxemia (19). To validate that these effects were *CX3CR1*-dependent, and to further characterize *CX3CR1*-variants in neurodegeneration and vascular damage in DR, we assessed the effect of LPS-mediated inflammation in the *hCX3CR1^I249/M280^* retina (**Figure 1**). Consistent with previously published studies in the diabetic retina in *CX3CR1*-KO mice, LPS-induced acute inflammation (4d LPS treatment) in *hCX3CR1^I249/M280^* expressing mice, revealed increased angiogenesis and abundant microglia clustering around the vasculature (**Figure 1**) (19). In addition to these pathological changes, RT-qPCR analysis showed significant increases in gene expression for proinflammatory genes in *hCX3CR1^I249/M280^* mice in comparison to LPS-treated *CX3CR1*-WT mice (**Figure 1**).

In the diabetic retina, vascular damage leads to leakage of serum proteins and danger associated molecular patterns (DAMPs) and a decrease in neuronal derived CX3CL1 (fractalkine), creating a loop of inflammation perpetuated by microglia pro-inflammatory cytokine production (14, 19, 44). Furthermore, diabetic *CX3CR1*-KO and *hCX3CR1^I249/M280^* mice were shown to exhibit greater neuronal cell loss, increased vascular damage, and elevated proinflammatory cytokine production (**Figures 4**, **5**) (19). In addition to a heightened proinflammatory response in diabetic *CX3CR1*-KO mice, previous studies showed that *CX3CR1*-KO microglia display a gene signature of premature ageing, with an increased expression of proinflammatory genes under naïve conditions that is further amplified with LPS treatment (45). We recently reported that transient microglia depletion and repopulation utilizing the genetic CX3CR1^CreER^:R26^iDTR^ model of microglia depletion prevented NeuN^+^RBPMS^+^ retinal ganglion cell and TUJ1^+^ axonal loss and alleviated vascular damage in the diabetic retina (26). This study revealed that in mice that retain functional CX3CR1-FKN signaling, microglia can be reprogrammed to be protective in the diabetic retina (26). However, CSF-1R antagonism in diabetic *CX3CR1*-KO and *hCX3CR1^I249/M280^* mice did not alleviate TUJ1^+^ axonal loss nor vascular damage in contrast to *CX3CR1*-WT mice (**Figures 4**, **5**). PLX-5622 treatment at doses used here does not induce 100% microglia ablation in *CX3CR1*-WT, *CX3CR1*-KO and *hCX3CR1^I249/M280^* mice (**Figures 3B, D**). Since microglia depletion was shown to reset the microglia gene signature to mirror the ND transcriptome, *CX3CR1*-WT microglia repopulate from a homeostatic microglia cell population that elicits neuro- and vasculo-protective effects in the diabetic retina (26). However, due to their underlying proinflammatory gene signature, *CX3CR1*-KO and *hCX3CR1^I249/M280^* microglia replenish from a retinal microglia population resistant to depletion that sustain a pro-inflammatory environment. Moreover, the morphology of microglia resistant to depletion was representative of amoeboid microglia with retracted cellular processes and larger cell bodies in all genotypes (**Figures 3C, E**). However, due to the protective effects PLX-5622 treatment caused in *CX3CR1*-WT mice, the morphology of microglia does not correlate to the neuro- and vasculo-protective effects of microglia. Together these data suggest that the *CX3CR1*-WT microglia population resistant to depletion acquire a homeostatic profile.

Previous studies revealed that *CX3CR1* deficiency in diabetic mice is associated with enhanced neuronal and axonal loss (14, 19). Four months diabetic *hCX3CR1^I249/M280^* mice were significantly more susceptible to NeuN^+^ neuronal cell death, microgliosis and astrogliosis in comparison to 4 months diabetic *CX3CR1*-WT mice (**Figure 2**). Consistent with exacerbated neuronal loss in 4 months diabetic *hCX3CR1^I249/M280^* mice, 10-weeks diabetic *CX3CR1*-KO and *hCX3CR1^I249/M280^* mice revealed significantly more TUJ1^+^ axonal loss in comparison to *CX3CR1*-WT mice (**Figures 4A, C**). PLX-5622 treatment in diabetic *CX3CR1*-WT mice prevented TUJ1^+^ axonal loss (**Figures 4A, C**). In contrast to these results, CSF-1R antagonism had no effect on TUJ1^+^ axonal loss in diabetic *CX3CR1*-KO and *hCX3CR1^I249/M280^* mice (**Figures 4A, C**). It is likely that the homeostatic phenotype of *CX3CR1*-WT microglia is responsible for production of neuroprotective mediators. We recently showed that CSF-1R antagonism in *CX3CR1*-WT mice was correlated with a significant reduction in expression of complement-associated genes and an increase in keratin gene expression, supporting the neuroprotective effects we see in PLX-5622 treated *CX3CR1*-WT mice (**Figures 4A, C**) (26). This neuro-protective gene expression in PLX-5622 treated *CX3CR1*-WT mice was sustained when microglia were allowed to repopulate, indicating that replenished *CX3CR1*-WT microglia retain a homeostatic gene profile (26). We hypothesized that the *CX3CR1*-deficient microglia that remain in the retina following PLX-5622 treatment, due to their proinflammatory profile will have limited capacity to support neuronal loss. Microglia repopulation was shown to be CX3CR1-FKN dependent (27). Microglial cells present in the retina after PLX-5622 treatment in *CX3CR1*-KO mice revealed a significant decrease in branch points, dendritic segment per cell and microglial repopulation-proliferation fewer rates (27). Intravitreal delivery of soluble CX3CL1 in the *CX3CR1*-HET retina significantly increased microglia repopulation-proliferation rates in PLX-5622 treated *CX3CR1*-HET mice, whereas CX3CL1 treatment in *CX3CR1*-KO mice had no effect on microglia repopulation-proliferation in PLX-5622 treated *CX3CR1*-KO retinas (27). Future studies to investigate the effects of microglia repopulation in the diabetic *CX3CR1*-KO and *hCX3CR1^I249/M280^* retina will further elucidate the role of FKN on microglial repopulation under diseased conditions. In addition to FKN-dependent repopulation, intravitreal FKN delivery in diabetic *FKN*-KO mice also reduces neuronal cell loss and Iba1^+^ microgliosis in the diabetic retina (19). Thus, the prevention of neuronal and axonal loss in PLX-5622 treated diabetic *CX3CR1*-WT reveals that CX3CR1-FKN signaling is necessary to induce neuroprotective cues in depletion-induced reprogramming of microglia.

Vasoregulation was shown to be altered in the early diabetic rat retina and was dependent on fractalkine-induced restriction at sites of microglial-capillary contact and pharmacological inhibition of *CX3CR1* prevented fractalkine-induced vaso-restriction (6). Our data complements these findings and revealed that PLX-5622 treated non-diabetic *CX3CR1*-KO and *hCX3CR1^I249/M280^* had an increase in fibrinogen deposition (**Figure 5**). In the diabetic *CX3CR1*-WT retina, CSF-1R antagonism reduced angiogenesis and fibrinogen deposition (**Figure 5**). PLX-5622 treatment in diabetic *CX3CR1*-KO mice had no effect on angiogenesis but led to a significant reduction in fibrinogen deposition (**Figure 5**). However, PLX-5622 treatment did not alleviate angiogenesis nor fibrinogen deposition in the diabetic *hCX3CR1^I249/M280^* retina (**Figure 5**). These findings reveal that receptor variants behave differently in terms of vascular pathology compared to *CX3CR1*-KO mice, indicating that *hCX3CR1^I249/M280^* mice provide a complementary model to study the CX3CR1-FKN signaling axis in regard to vascular abnormalities.

To further validate the findings in *hCX3CR1^I249/M280^* mice and to ensure that the observed effects in *hCX3CR1^I249/M280^* mice were not due to the presence of human *CX3CR1* in the mouse loci, we characterized the effects of either the I250 (*mCX3CR1^I250/WT^*) or M281 (*mCX3CR1^M281/WT^*) polymorphism in the mouse *CX3CR1* loci in the diabetic murine retina (**Supplementary Figures 3**-**6**). The phenotype in these mice (*mCX3CR1^I250/WT^* and *mCX3CR1^M281/WT^*) closely resembled the phenotype of the humanized model *hCX3CR1^I249/M280^* mice. Surprisingly, PLX-5622 treatment in diabetic m*CX3CR1^I250/WT^* and m*CX3CR1^M281/WT^* led to a 100% depletion of Iba1^+^ cells in the retina (**Supplementary Figure 3B–D**). These findings indicate that the presence of these polymorphisms strongly affects retinal microglial sensitivity to CSF-1R blockade.

Overall, this study reveals that the protective effects of microglia depletion in *CX3CR1*-WT mice are *CX3CR1*-dependent as microglia depletion in *CX3CR1*-KO and *hCX3CR1^I249/M280^* mice did not alleviate retinal degeneration. We characterized the humanized *hCX3CR1^I249/M280^*-expressing mice for the first time in the murine STZ model of diabetes. Our data revealed that PLX-5622 treated *CX3CR1*-KO microglial vascular responses diverge from *hCX3CR1^I249/M280^*, m*CX3CR1^I250/WT^* and m*CX3CR1^M281/WT^* microglial responses. These findings indicate that utilizing *hCX3CR1^I249/M280^* mice in studies assessing CX3CR1-FKN signaling effects on the vasculature, represent a complementary model to study dysregulated CX3CR1-FKN signaling. Strategies to alter microglial densities in the human population may not be beneficial as we did not detect neuro- or vasculo-protection in PLX-5622 treated *hCX3CR1^I249/M280^*, *mCX3CR1^I250/WT^* and *mCX3CR1^M281/WT^* mice. Future studies to analyze the transcriptional changes of homeostatic, and repopulated microglia from *CX3CR1*-WT, *CX3CR1*-KO, *FKN*-KO and *hCX3CR1^I249/M280^* mice will be valuable to further define the mechanisms by which FKN exerts its regulatory roles in the diabetic retina.

## Data availability statement

The original contributions presented in the study are included in the article/supplementary material, further inquiries can be directed to the corresponding author.

## Ethics statement

The animal study was reviewed and approved by University of Texas at San Antonio Institutional Animal Care and Use Committee. Written informed consent was obtained from the owners for the participation of their animals in this study.

## Author contributions

AEC developed the concept of the study. AEC and KC designed experiments, analyzed and interpreted data and wrote the manuscript. Research development and data acquisition was performed by KC, DR, AM, DV, IG, IT, AA, PV, and SC. SG, AC, RR, and TK interpreted data and advised in manuscript preparation. All authors contributed to the article and approved the submitted version.

## Supplementary material

Supplementary Figure 1 Acute LPS-induced inflammation in the *hCX3CR1^I249/M280^* retina. **(A)** Schematic displaying the experimental method used to image whole retinas in all the studies presented. Retinas were divided into 4 sections, each containing central, medial and peripheral regions of the retina. Two images per retinal region were acquired for a total of 6 images per retina. **(B, D)**, *hCX3CR1^I249/M280^* mice were i.p. injected with 0.08mg/Kg LPS once daily for four days (4d), and naïve age-matched controls received PBS. Graphical representation for RT-qPCR analysis of retinas for relative mRNA expression for *Il1β* **(B)**, *Il6* **(C)** and *Nos2* **(D)**. Data show mean ± SD, *n* = 3 mice per group where each dot represents an individual mouse. **P*<0.05, ****P*<0.001 using Student’s *t*-test, with Welch’s correction.

Supplementary Figure 2 PLX-5622 treatment does not prevent TUJ1^+^ axonal loss or vascular damage in the *CX3CR1*-KO and *hCX3CR1^I249/M280^* retina. **(A)** Experimental design to pharmacologically deplete microglia in non-diabetic and 8-wks diabetic *CX3CR1*-WT, *CX3CR1*-KO, and *hCX3CR1^I249/M280^* mice for two weeks. Non-diabetic control mice received citrate buffer. Non-depleted, non-diabetic and diabetic controls remained on normal chow. **(B, C)**, Merged confocal images of retinal tissues stained for TUJ1 (turquoise) and GFAP (magenta) **(B)** and CD31 (red) and fibrinogen (green) **(C)** in *CX3CR1*-WT, *CX3CR1*-KO and *hCX3CR1^I249/M280^* mice. Confocal images represent the peripheral retina. Scale bars measure 50µm.

Supplementary Figure 3 CSF-1R antagonism induces 100% microglia depletion in the *mCX3CR1^I250/WT^* and *mCX3CR1^M281/WT^* diabetic retina. Microglia were pharmacologically depleted using PLX-5622 in non-diabetic and 8-wks diabetic *CX3CR1*-WT, *mCX3CR1^I250/WT^* and *mCX3CR1^M281/WT^* mice for two weeks. Non-diabetic control mice received citrate buffer. Non-depleted, non-diabetic and diabetic controls remained on normal chow. Confocal images of retinal tissues stained for Iba1 (green) **(A)** and transformation index cellular tracings **(B)** in *CX3CR1*-WT, *mCX3CR1^I250/WT^* and *mCX3CR1^M281/WT^* mice. Confocal images represent the retinal ganglion cell layer in the peripheral retina. **(C, D)**, Quantification of retinal IHC analysis for Iba1^+^ cells/mm^3^ **(C)** and transformation index **(D)**. Data show the average of the 2 central, 2 medial and 2 peripheral images taken per mouse. Data show mean ± SD, *n* = 6 to 10 mice per group where each dot represents an individual mouse **(C)**. Transformation index data show mean ± SD *n* = 52 to 150 microglia per group for *n*=5 mice where each dot represents an individual microglia cell **(D)**. **P*<0.05, ***P*<0.01, ****P*<0.001 *****P*<0.0001 using 2-way ANOVA. Scale bars measure 50µm.

Supplementary Figure 4 PLX-5622 treatment does not prevent TUJ1^+^ axonal loss in the diabetic *mCX3CR1^I250/WT^* and *mCX3CR1^M281/WT^* retina. Microglia were pharmacologically depleted using PLX-5622 in non-diabetic and 8-wks diabetic *CX3CR1*-WT, *mCX3CR1^I250/WT^* and *mCX3CR1^M281/WT^* mice for two weeks. Non-diabetic control mice received citrate buffer. Non-depleted, non-diabetic and diabetic controls remained on normal chow. Confocal images of retinal tissues stained for TUJ1 (turquoise) **(A)** and GFAP (magenta) **(B)** in *CX3CR1*-WT, *mCX3CR1^I250/WT^* and *mCX3CR1^M281/WT^* mice. Confocal images represent the retinal ganglion cell layer in the peripheral retina. **(C, D)**, Quantification of retinal IHC analysis for TUJ1^+^ percent immunoreactive area **(C)** and GFAP^+^ percent immunoreactive area **(D)**. Data show the average of the 2 central, 2 medial and 2 peripheral images taken per mouse. Data show mean ± SD, *n* = 4 to 10 mice per group where each dot represents an individual mouse. **P*<0.05, ***P*<0.01, ****P*<0.001 *****P*<0.0001 using 2-way ANOVA. Scale bars measure 50µm.

Supplementary Figure 5 PLX-5622 treatment does not alleviate vascular damage in the diabetic *mCX3CR1^I250/WT^* and *mCX3CR1^M281/WT^* retina. Microglia were pharmacologically depleted using PLX-5622 in non-diabetic and 8-wks diabetic *CX3CR1*-WT, *mCX3CR1^I250/WT^* and *mCX3CR1^M281/WT^* mice for two weeks. Non-diabetic control mice received citrate buffer. Non-depleted, non-diabetic and diabetic controls remained on normal chow. Confocal images of retinal tissues stained for CD31 (red) **(A)** and fibrinogen (white) **(B)** in *CX3CR1*-WT, *mCX3CR1^I250/WT^* and *mCX3CR1^M281/WT^* mice. Confocal images represent the retinal ganglion cell layer in the peripheral retina. **(C, D)**, Quantification of retinal IHC analysis for CD31^+^ percent immunoreactive area **(C)** and fibrinogen^+^ percent immunoreactive area **(D)**. Data show the average of the 2 central, 2 medial and 2 peripheral images taken per mouse. Data show mean ± SD, *n* = 4 to 10 mice per group where each dot represents an individual mouse. **P*<0.05, ***P*<0.01, ****P*<0.001 *****P*<0.0001 using 2-way ANOVA. Scale bars measure 50µm.

Supplementary Figure 6 PLX-5622 treatment does not prevent TUJ1^+^ axonal loss or vascular damage in the diabetic *mCX3CR1^I250/WT^* and *mCX3CR1^M281/WT^* retina. **(A)** Experimental design to pharmacologically deplete microglia in non-diabetic and 8-wks diabetic *CX3CR1*-WT, *mCX3CR1^I250/WT^* and *mCX3CR1^M281/WT^* mice for two weeks. Non-diabetic control mice received citrate buffer. Non-depleted, non-diabetic and diabetic controls remained on normal chow. **(B, C)**, Merged confocal images of retinal tissues stained for TUJ1 (turquoise) and GFAP (magenta) **(B)** and CD31 (red) and fibrinogen (green) **(C)** in *CX3CR1*-WT, *mCX3CR1^I250/WT^* and *mCX3CR1^M281/WT^* mice. Confocal images represent the peripheral retina. Scale bars measure 50µm.

## Glossary

**Table:**

CD31	pecam-1 CX3CL1
FKN	fractalkine
CX3CR1-KO	knock-out transgenic CX3CR1-GFP reporter mice CX3CR1GFP/GFP
DR	diabetic retinopathy
EAE	experimental autoimmune encephalomyelitis;
GFAP	glial fibrillary acidic protein
HBSS	hank’s balanced salt solution
hCX3CR1I249/M280	human polymorphic variant of the CX3CR1 gene with amino acid substitutions at residues 249 (valine substituted for isoleucine) and 280 (threonine substituted for methionine)
Iba1	ionized calcium binding adaptor molecule-1
i.p.	intra-peritoneal
LPS	lipopolysaccharide
mCX3CR1I250	transgenic mice expressing polymorphic variant of CX3CR1 gene with amino acid substitution at 250 residue (valine substituted for isoleucine) mCX3CR1M281 transgenic mice expressing polymorphic variant of CX3CR1 gene with amino acid substitution at 281 residue (threonine substituted for methionine)
NeuN	neuronal nuclei
PFA	paraformaldehyde
ROS	reactive oxygen species
STZ	streptozotocin
TUJ1	b tubulin III
VEGF	vascular endothelial factor
