# Supplementary figures and images for: Pharmacological depletion of microglia alleviates neuronal and vascular damage in the diabetic CX3CR1-WT retina but not in CX3CR1-KO or hCX3CR1I249/M280-expressing retina
Source: Front Immunol. 2023 Mar 22;14:1130735. doi: 10.3389/fimmu.2023.1130735 (PMC10077890; doi:10.3389/fimmu.2023.1130735)

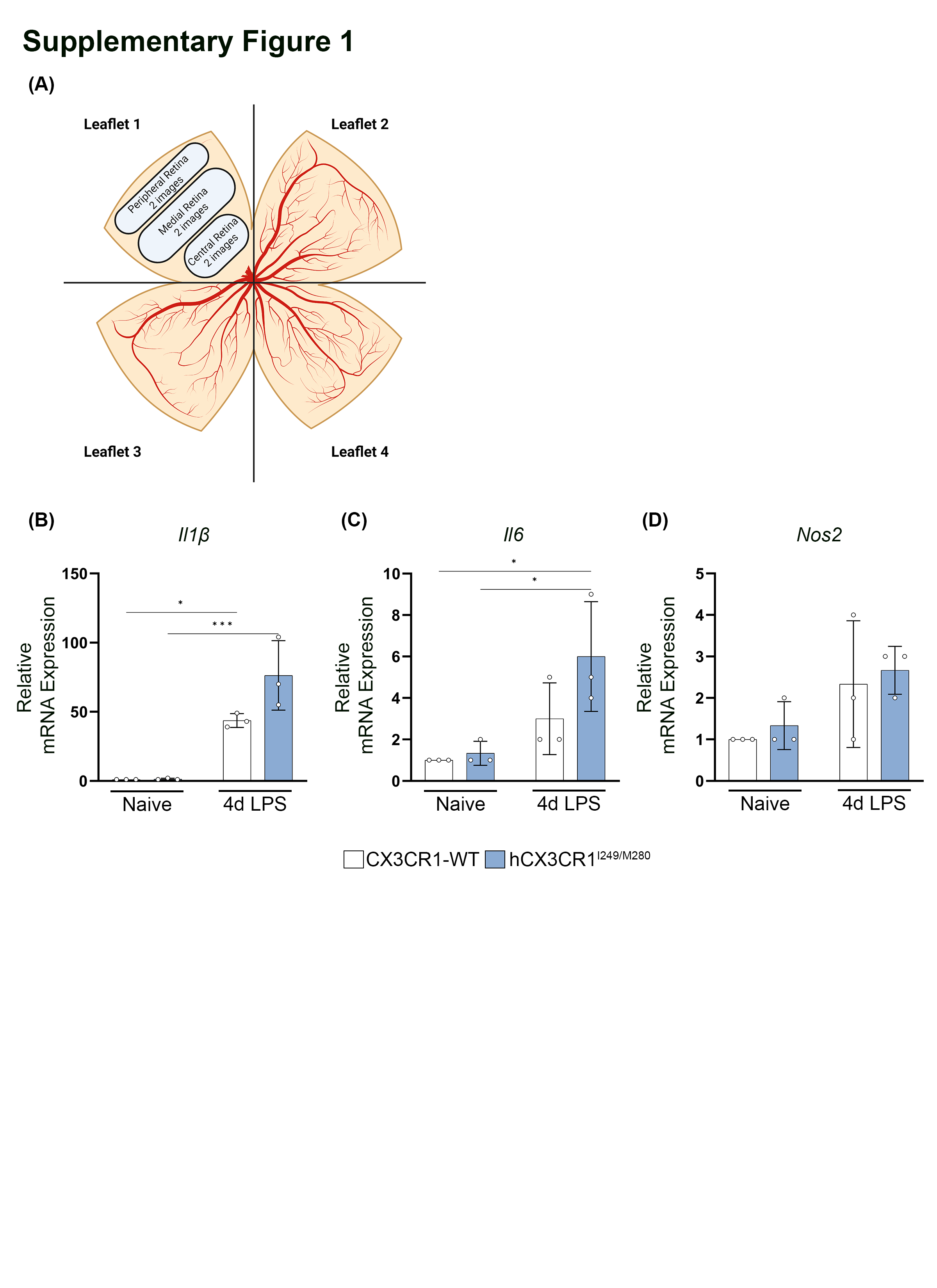

Supplement: Supplementary Figure 1 — Acute LPS-induced inflammation in the hCX3CR1I249/M280 retina. (A) Schematic displaying the experimental method used to image whole retinas in all the studies presented. Retinas were divided into 4 sections, each containing central, medial and peripheral regions of the retina. Two images per retinal region were acquired for a total of 6 images per retina. (B, D), hCX3CR1I249/M280 mice were i.p. injected with 0.08mg/Kg LPS once daily for four days (4d), and naïve age-matched controls received PBS. Graphical representation for RT-qPCR analysis of retinas for relative mRNA expression for Il1β (B), Il6 (C) and Nos2 (D). Data show mean ± SD, n = 3 mice per group where each dot represents an individual mouse. *P<0.05, ***P<0.001 using Student’s t-test, with Welch’s correction. [file Image_1.tif]

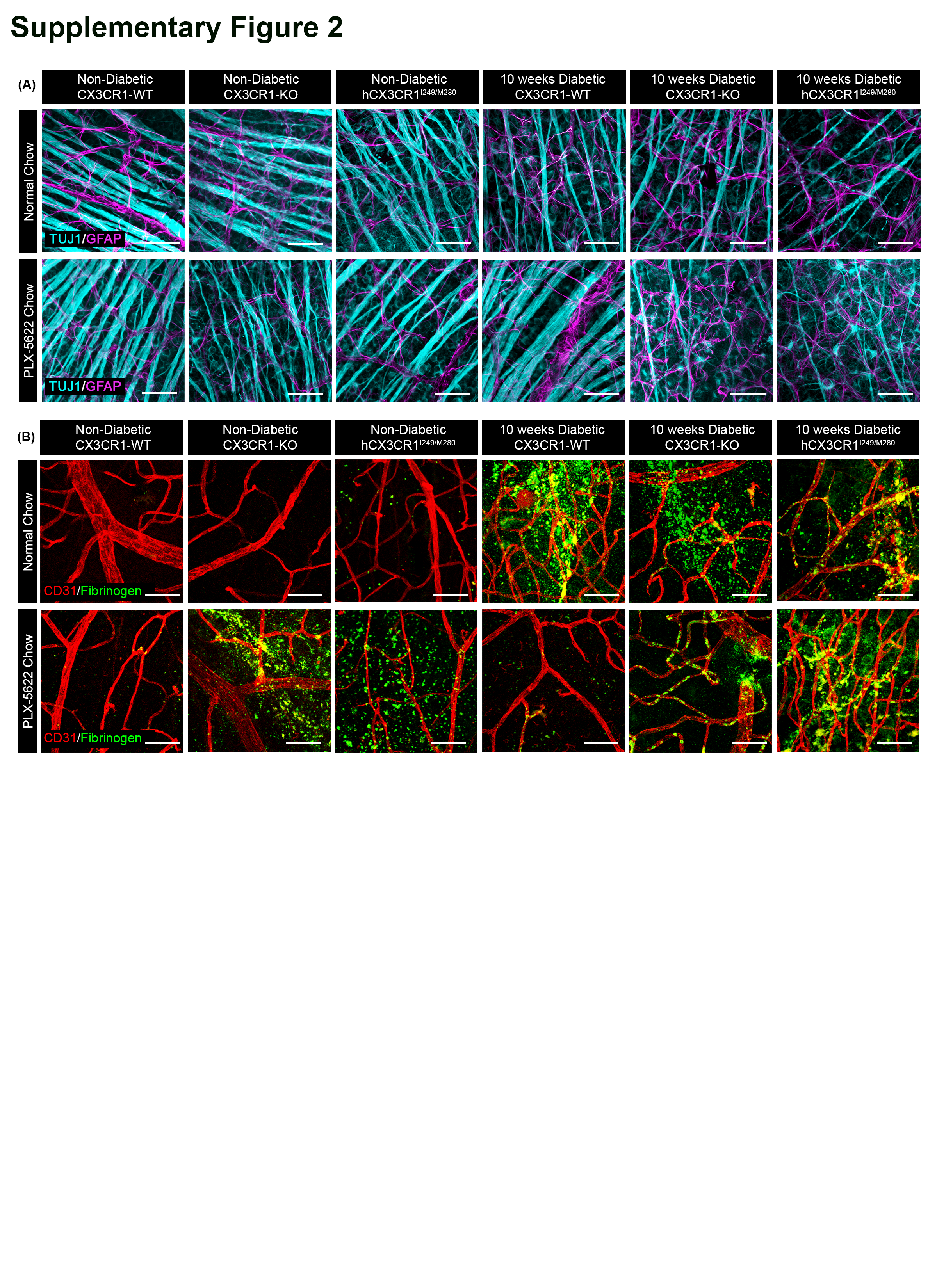

Supplement: Supplementary Figure 2 — PLX-5622 treatment does not prevent TUJ1+ axonal loss or vascular damage in the CX3CR1-KO and hCX3CR1I249/M280 retina. (A) Experimental design to pharmacologically deplete microglia in non-diabetic and 8-wks diabetic CX3CR1-WT, CX3CR1-KO, and hCX3CR1I249/M280 mice for two weeks. Non-diabetic control mice received citrate buffer. Non-depleted, non-diabetic and diabetic controls remained on normal chow. (B, C), Merged confocal images of retinal tissues stained for TUJ1 (turquoise) and GFAP (magenta) (B) and CD31 (red) and fibrinogen (green) (C) in CX3CR1-WT, CX3CR1-KO and hCX3CR1I249/M280 mice. Confocal images represent the peripheral retina. Scale bars measure 50µm. [file Image_2.tif]

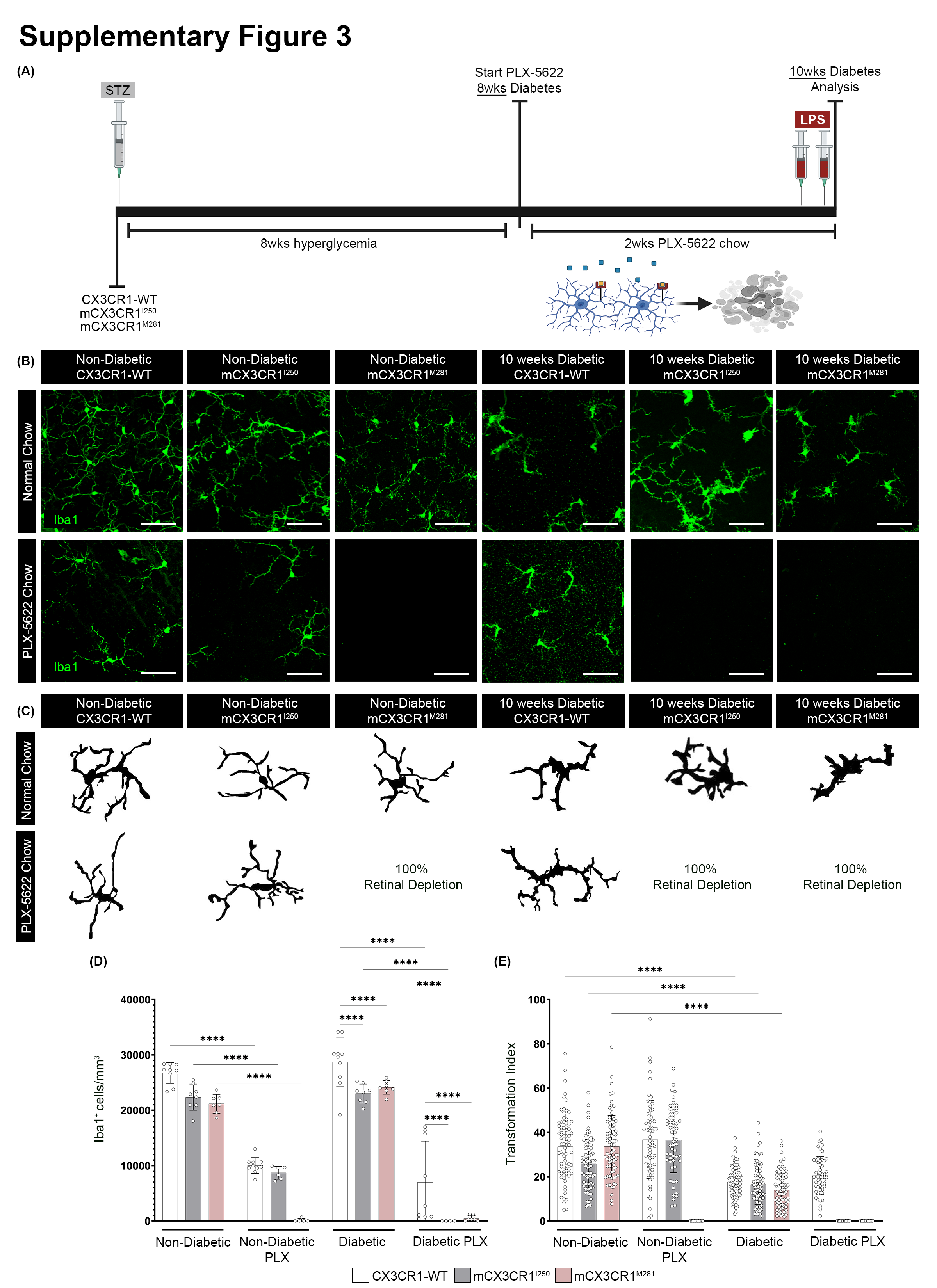

Supplement: Supplementary Figure 3 — CSF-1R antagonism induces 100% microglia depletion in the mCX3CR1I250/WT and mCX3CR1M281/WT diabetic retina. Microglia were pharmacologically depleted using PLX-5622 in non-diabetic and 8-wks diabetic CX3CR1-WT, mCX3CR1I250/WT and mCX3CR1M281/WT mice for two weeks. Non-diabetic control mice received citrate buffer. Non-depleted, non-diabetic and diabetic controls remained on normal chow. Confocal images of retinal tissues stained for Iba1 (green) (A) and transformation index cellular tracings (B) in CX3CR1-WT, mCX3CR1I250/WT and mCX3CR1M281/WT mice. Confocal images represent the retinal ganglion cell layer in the peripheral retina. (C, D), Quantification of retinal IHC analysis for Iba1+ cells/mm3 (C) and transformation index (D). Data show the average of the 2 central, 2 medial and 2 peripheral images taken per mouse. Data show mean ± SD, n = 6 to 10 mice per group where each dot represents an individual mouse (C). Transformation index data show mean ± SD n = 52 to 150 microglia per group for n=5 mice where each dot represents an individual microglia cell (D). *P<0.05, **P<0.01, ***P<0.001 ****P<0.0001 using 2-way ANOVA. Scale bars measure 50µm. [file Image_3.tif]

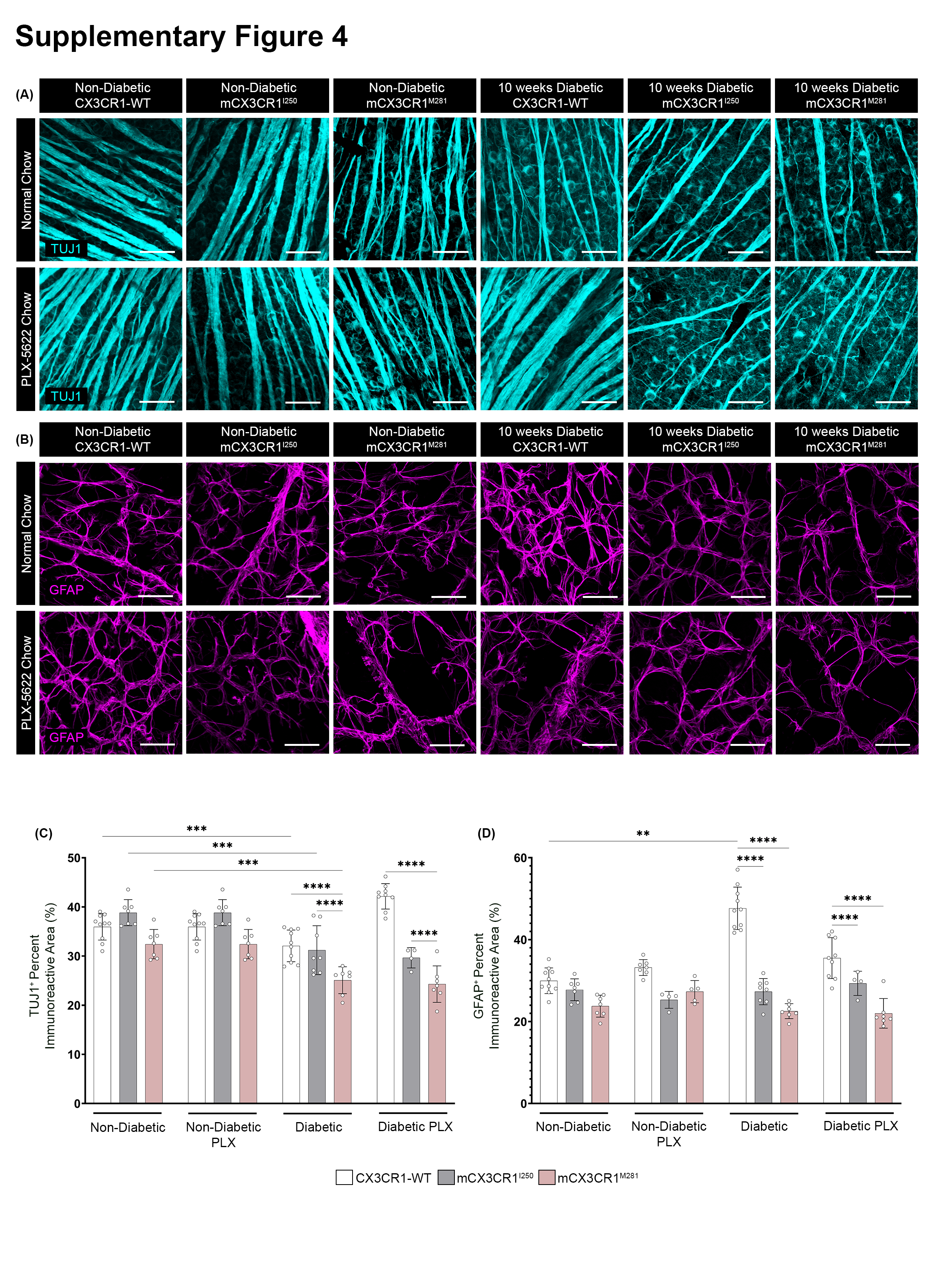

Supplement: Supplementary Figure 4 — PLX-5622 treatment does not prevent TUJ1+ axonal loss in the diabetic mCX3CR1I250/WT and mCX3CR1M281/WT retina. Microglia were pharmacologically depleted using PLX-5622 in non-diabetic and 8-wks diabetic CX3CR1-WT, mCX3CR1I250/WT and mCX3CR1M281/WT mice for two weeks. Non-diabetic control mice received citrate buffer. Non-depleted, non-diabetic and diabetic controls remained on normal chow. Confocal images of retinal tissues stained for TUJ1 (turquoise) (A) and GFAP (magenta) (B) in CX3CR1-WT, mCX3CR1I250/WT and mCX3CR1M281/WT mice. Confocal images represent the retinal ganglion cell layer in the peripheral retina. (C, D), Quantification of retinal IHC analysis for TUJ1+ percent immunoreactive area (C) and GFAP+ percent immunoreactive area (D). Data show the average of the 2 central, 2 medial and 2 peripheral images taken per mouse. Data show mean ± SD, n = 4 to 10 mice per group where each dot represents an individual mouse. *P<0.05, **P<0.01, ***P<0.001 ****P<0.0001 using 2-way ANOVA. Scale bars measure 50µm. [file Image_4.tif]

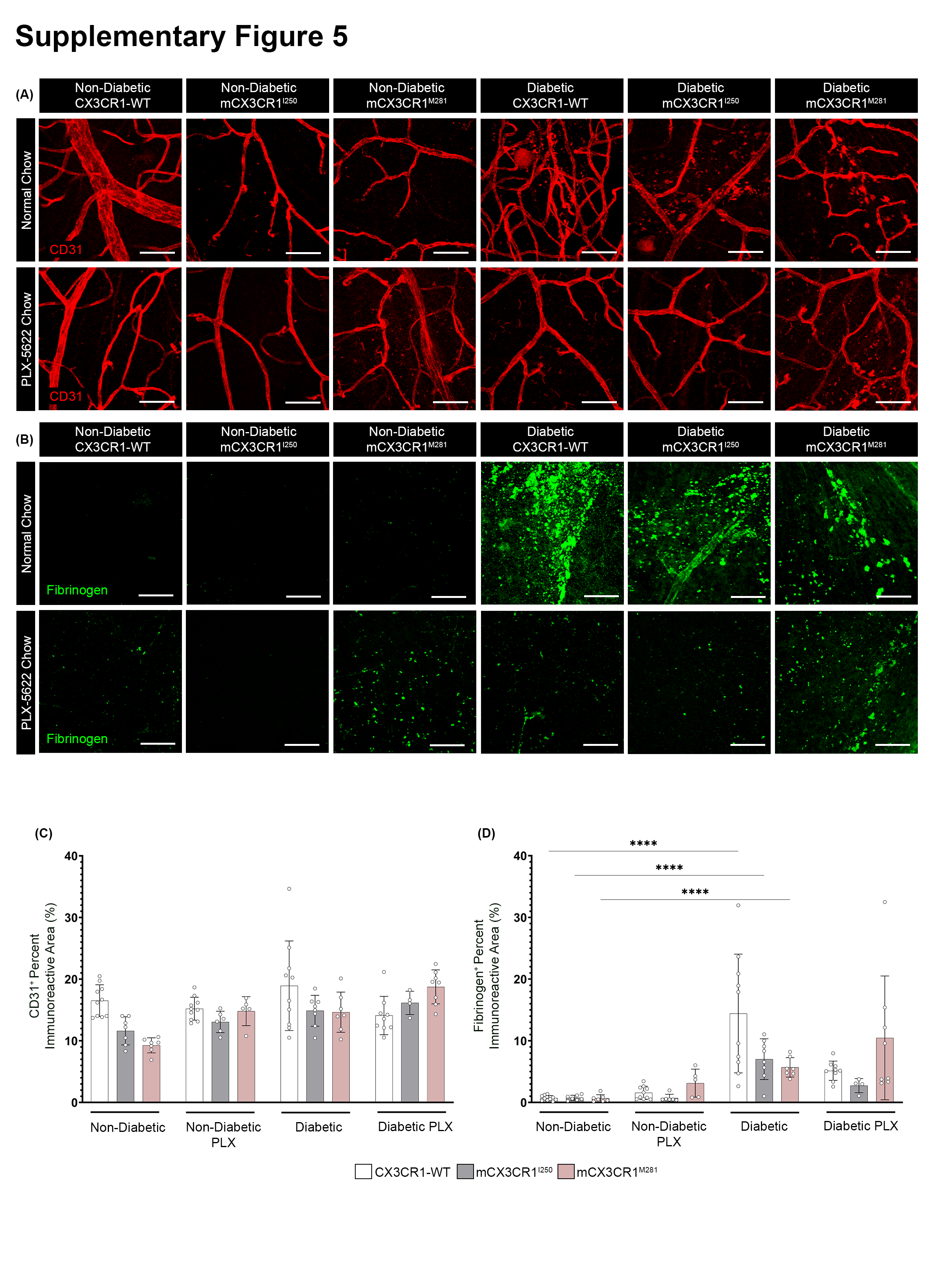

Supplement: Supplementary Figure 5 — PLX-5622 treatment does not alleviate vascular damage in the diabetic mCX3CR1I250/WT and mCX3CR1M281/WT retina. Microglia were pharmacologically depleted using PLX-5622 in non-diabetic and 8-wks diabetic CX3CR1-WT, mCX3CR1I250/WT and mCX3CR1M281/WT mice for two weeks. Non-diabetic control mice received citrate buffer. Non-depleted, non-diabetic and diabetic controls remained on normal chow. Confocal images of retinal tissues stained for CD31 (red) (A) and fibrinogen (white) (B) in CX3CR1-WT, mCX3CR1I250/WT and mCX3CR1M281/WT mice. Confocal images represent the retinal ganglion cell layer in the peripheral retina. (C, D), Quantification of retinal IHC analysis for CD31+ percent immunoreactive area (C) and fibrinogen+ percent immunoreactive area (D). Data show the average of the 2 central, 2 medial and 2 peripheral images taken per mouse. Data show mean ± SD, n = 4 to 10 mice per group where each dot represents an individual mouse. *P<0.05, **P<0.01, ***P<0.001 ****P<0.0001 using 2-way ANOVA. Scale bars measure 50µm. [file Image_5.tif]

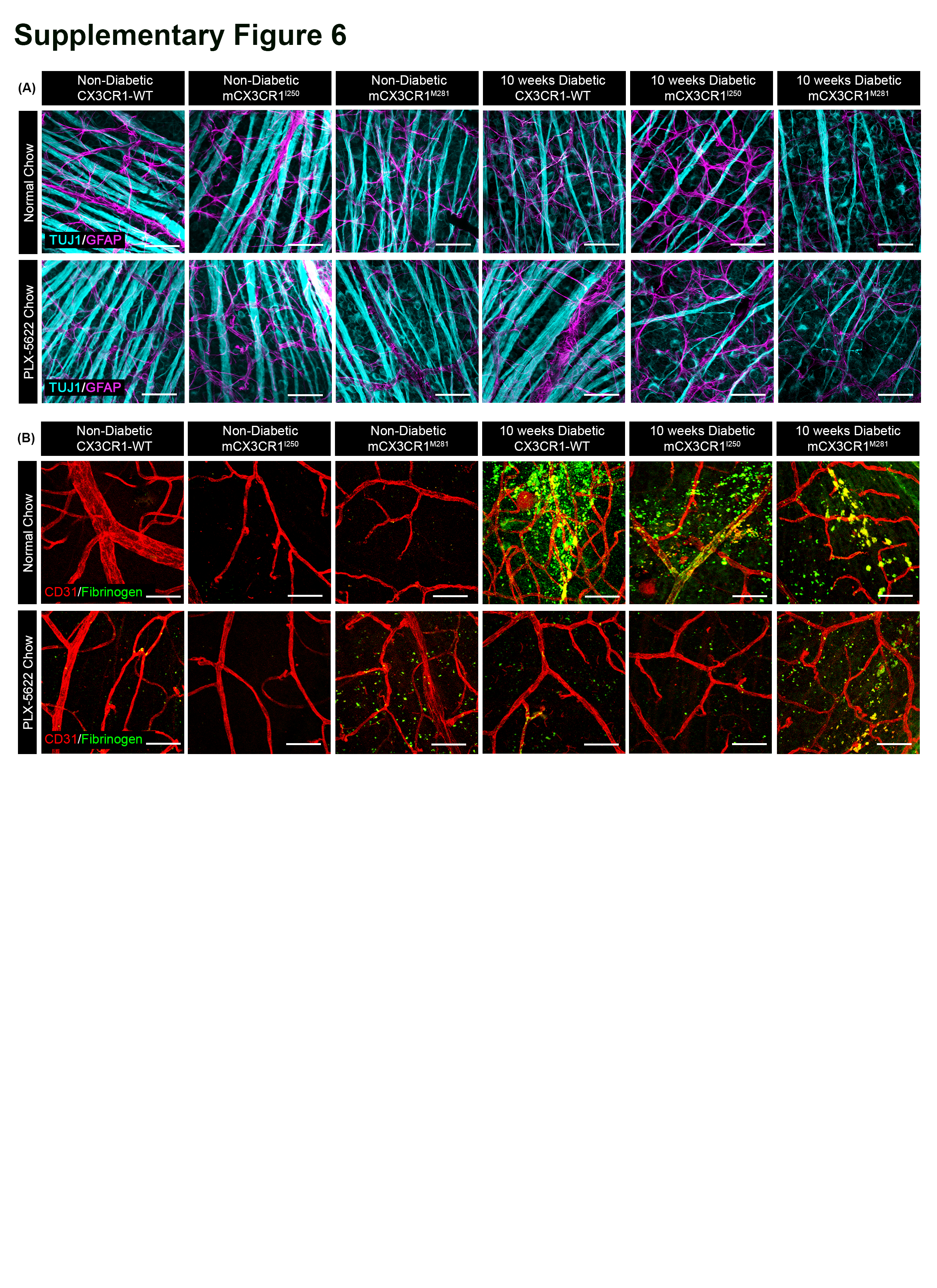

Supplement: Supplementary Figure 6 — PLX-5622 treatment does not prevent TUJ1+ axonal loss or vascular damage in the diabetic mCX3CR1I250/WT and mCX3CR1M281/WT retina. (A) Experimental design to pharmacologically deplete microglia in non-diabetic and 8-wks diabetic CX3CR1-WT, mCX3CR1I250/WT and mCX3CR1M281/WT mice for two weeks. Non-diabetic control mice received citrate buffer. Non-depleted, non-diabetic and diabetic controls remained on normal chow. (B, C), Merged confocal images of retinal tissues stained for TUJ1 (turquoise) and GFAP (magenta) (B) and CD31 (red) and fibrinogen (green) (C) in CX3CR1-WT, mCX3CR1I250/WT and mCX3CR1M281/WT mice. Confocal images represent the peripheral retina. Scale bars measure 50µm. [file Image_6.tif]
