# Supplementary material for: Pharmacological depletion of microglia alleviates neuronal and vascular damage in the diabetic CX3CR1-WT retina but not in CX3CR1-KO or hCX3CR1I249/M280-expressing retina
Source: Front Immunol. 2023 Mar 22;14:1130735. doi: 10.3389/fimmu.2023.1130735 (PMC10077890; doi:10.3389/fimmu.2023.1130735)
Supplement: Supplementary file 7 [file Table_1.pdf]

Supplementary Table 1. Information for the primary antibody combinations and species-specific secondary antibodies used for immunofluorescent analysis.

Iba1-NeuN Cocktail					
Primary	RRID	Concentration	Secondary	RRID	Concentration
Rabbit anti-ionized calcium binding adaptor molecule-1 (Iba1)	AB_839504	1:3000	Donkey anti-Rabbit 488	AB_2313584	1:1000
Mouse anti-neuronal nuclei (NeuN)	AB_2298772	1:4000	Goat anti-Mouse Cy3	AB_2338709	1:1000
TUJ1-GFAP Cocktail					
Primary	RRID	Concentration	Secondary	RRID	Concentration
Mouse anti- beta tubulin III (TUJ1)	AB_10063408	1:1000	Goat anti-Mouse Cy3	AB_2338709	1:1000
Rat anti-glial fibrillary acidic protein (GFAP)	AB_2532994	1:4000	Donkey anti-Rat Cy5	AB_2340694	1:1000
CD31-Fibrinogen Cocktail					
Primary	RRID	Concentration	Secondary	RRID	Concentration
Rat anti-platelet endothelial cell adhesion molecule (PECAM-1/CD-31)	AB_393571	1:500	Goat anti-Rat Cy3	AB_2338394	1:1000
Rabbit anti-fibrinogen	AB_578481	1:2000	Goat anti-rabbit Cy5	AB_2338078	1:1000
